# Supplementary material for: A quadri-fluorescence SARS-CoV-2 pseudovirus system for efficient antigenic characterization of multiple circulating variants
Source: Cell Rep Methods. 2024 Sep 6;4(9):100856. doi: 10.1016/j.crmeth.2024.100856 (PMC11440059; doi:10.1016/j.crmeth.2024.100856)
Supplement: Document S1. Figures S1‒S5 and Tables S1, S2, and S4 [file mmc1.pdf]

Cell Reports Methods, Volume *4*

Supplemental information

A quadri-fluorescence SARS-CoV-2 pseudovirus system for efficient antigenic characterization of multiple circulating variants

Jijing Chen (陈积璟), Zehong Huang (黄泽宏), Jin Xiao (肖瑾), Shuangling Du (杜双伶), Qingfang Bu (布庆芳), Huilin Guo (郭慧琳), Jianghui Ye (叶江辉), Shiqi Chen (陈诗琦), Jiahua Gao (高佳华), Zonglin Li (李宗霖), Miaolin Lan (蓝妙琳), Shaojuan Wang (王邵娟), Tianying Zhang (张天英), Jiming Zhang (张继明), Yangtao Wu (巫洋涛), Yali Zhang (张雅丽), Ningshao Xia (夏宁邵), Quan Yuan (袁权), and Tong Cheng (程通)

Supplementary Figures

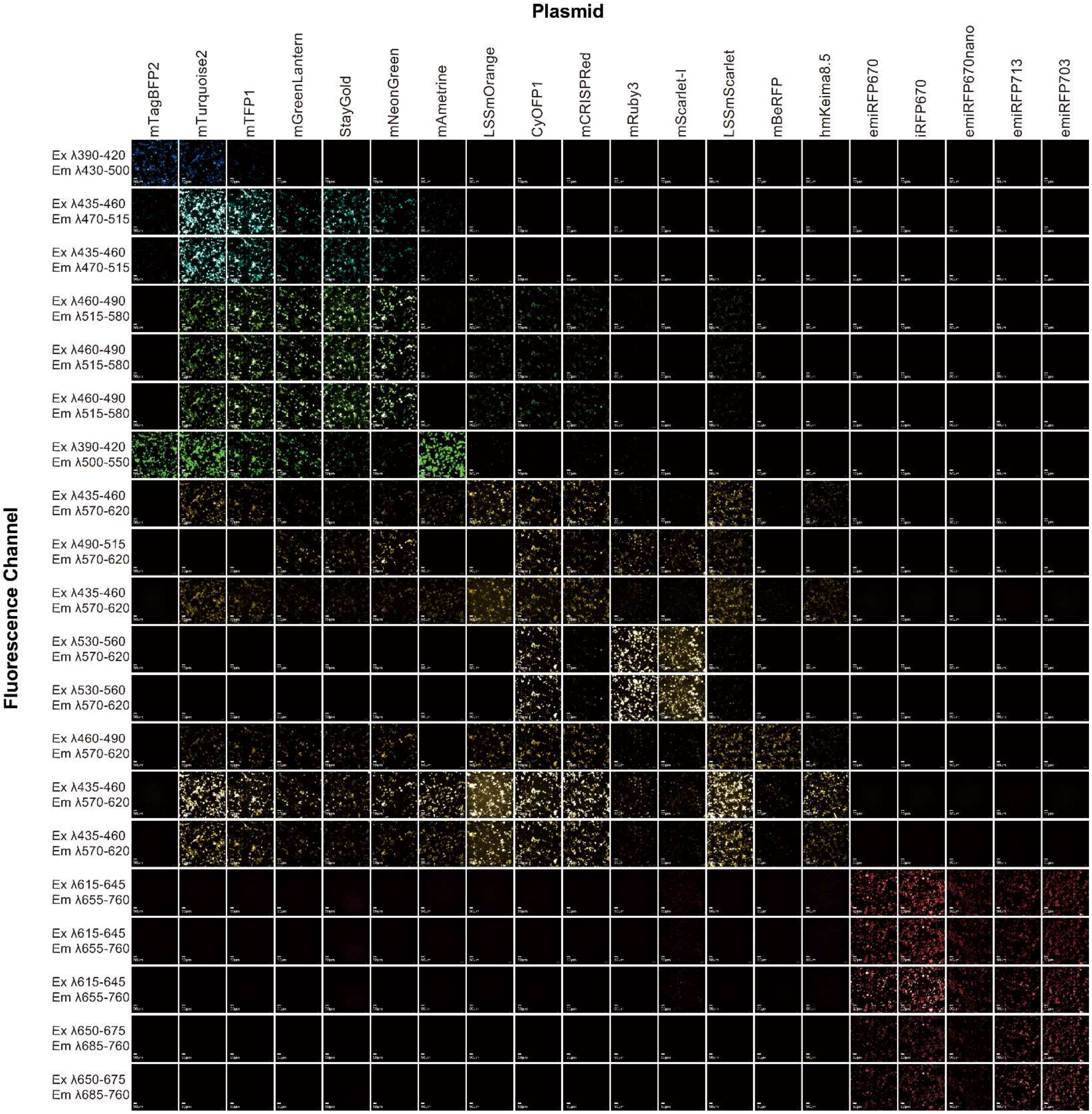

Figure S1 | The fluorescence imaging of 20 fluorescent proteins used for screening in different fluorescence channels, related to the middle panel of Figure 1A.

A field-of-view of fluorescence imaging of 293T/17 cells transfected with each fluorescent plasmid for 48h. The left side is marked with the excitation wavelength (Ex) and emission wavelength (Em) corresponding to each fluorescence channel.

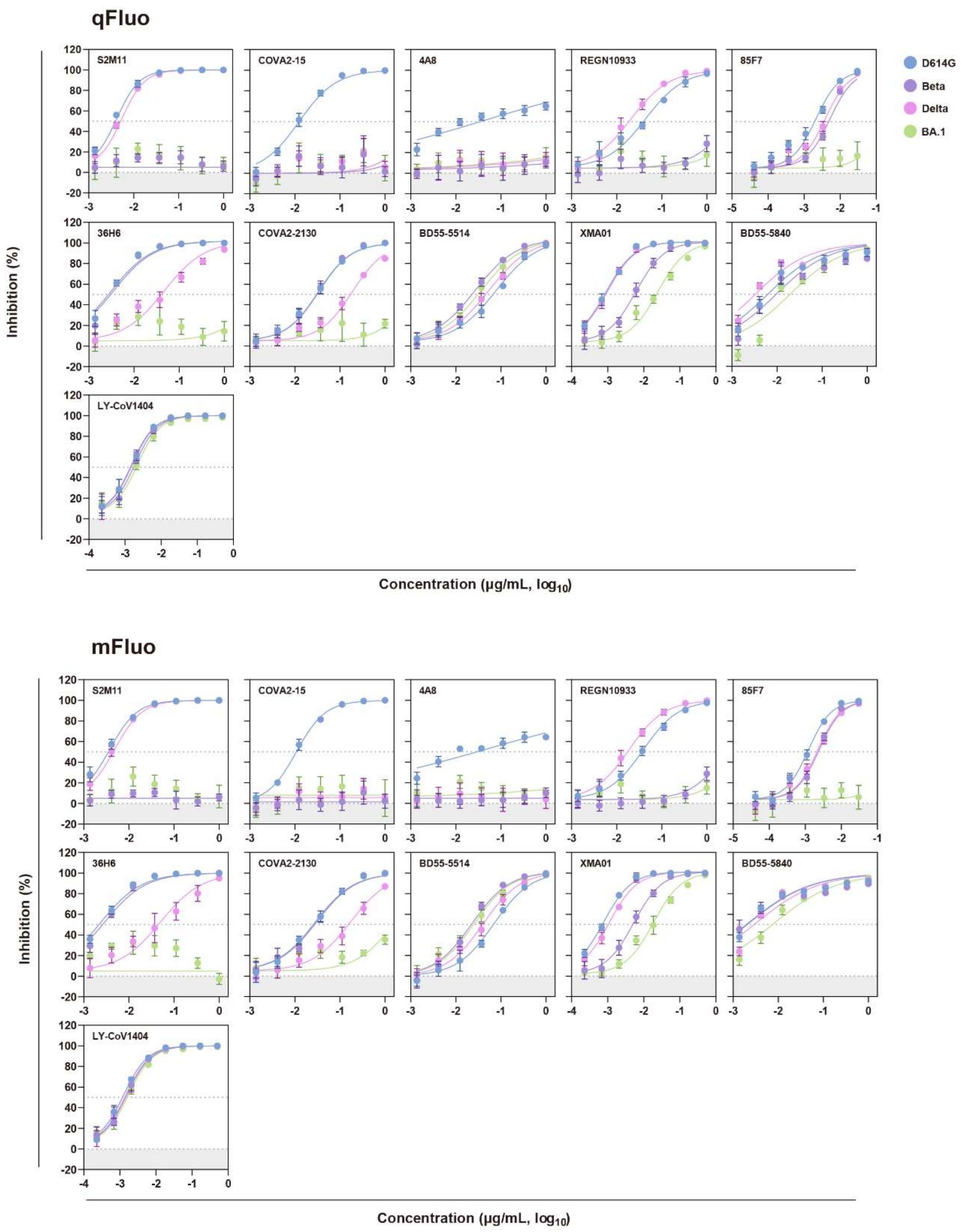

Figure S2 | Neutralization profiles for 11 mAbs detected by qFluo and mFluo systems, related to the left panel of Figure 4A.

All mAbs were tested at 3-fold serial dilutions. The data were plotted as the mean value and SD of ≥3 technical replicates.

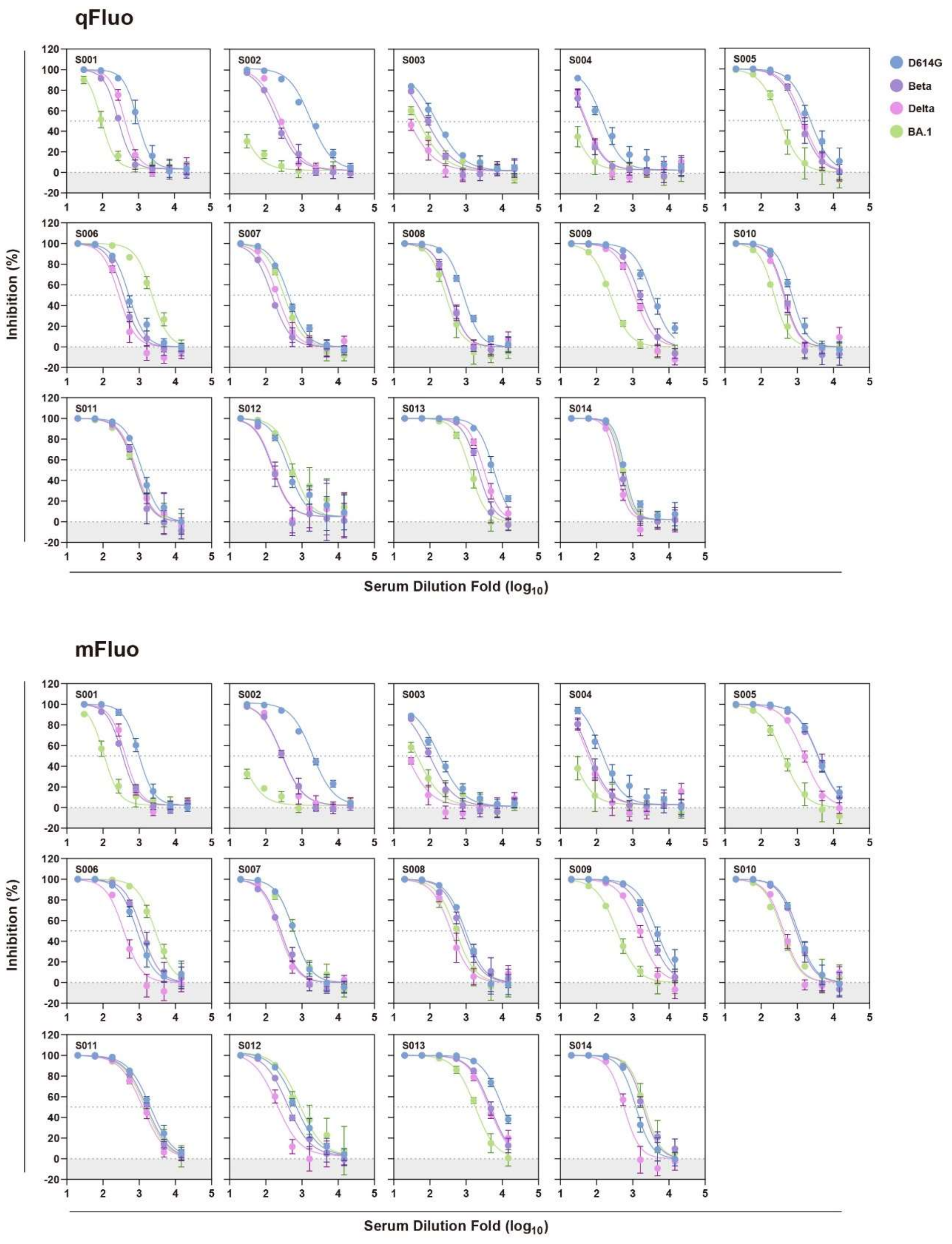

Figure S3 | Neutralization profiles for 14 plasmas detected by qFluo and mFluo systems, related to the right panel of Figure 4A.

All sera were tested at 3-fold serial dilutions. The data were plotted as the mean value and SD of ≥3 technical replicates.

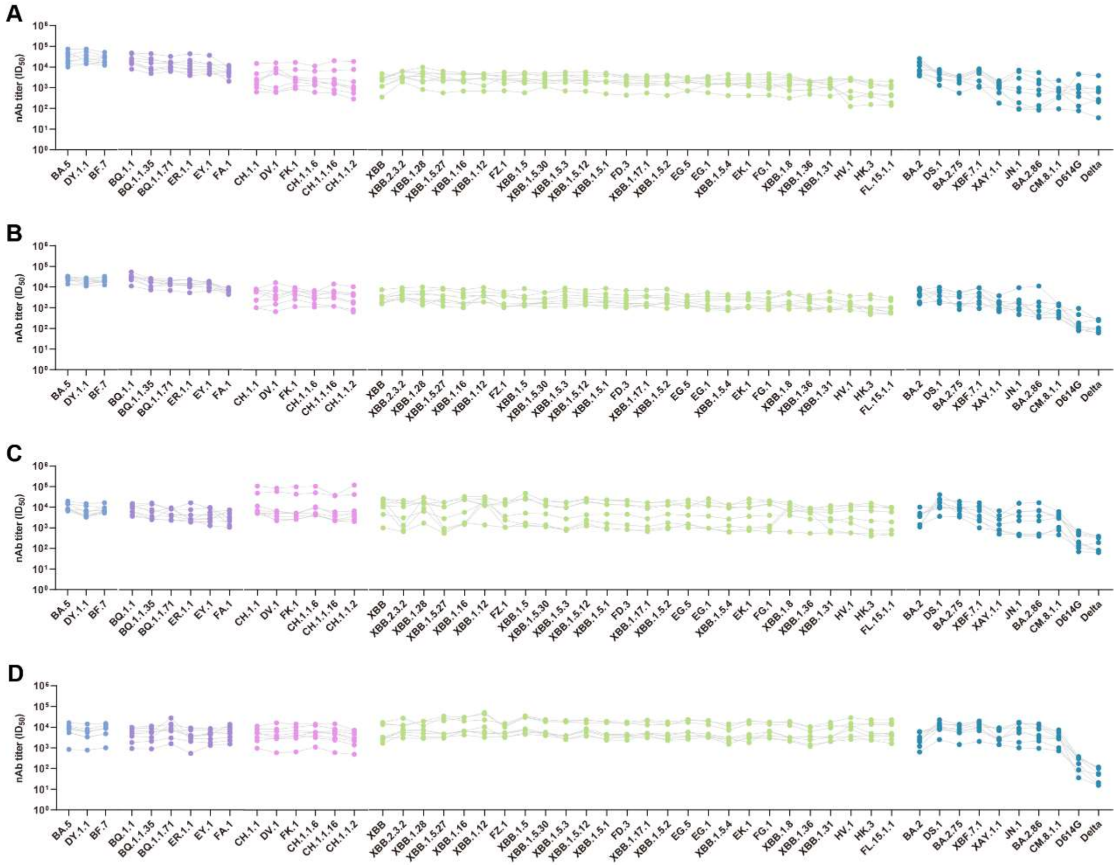

Figure S4 | Neutralizing antibody response profiles of immunized hamsters' sera, related to Figure 5.

Neutralization of 51 SARS-CoV-2 spike variants by hamsters' sera immunized with BA.5 (**A**, n=8), BQ.1.1 (**B**, n=8), CH.1.1 (**C**, n=8), and XBB.1.5 (**D**, n=8) antigens.

	NTD																															RBD																																	
	16	T19	R21	L24	P25	P26	A27	S50	Q52	H69	V70	V83	V127	G142	Y144	H146	K147	W152	E156	F157	R158	E180	Q183	I210	N211	L212	V213	L216	H245	A246	G252	G257	I332	G339	R346	K356	L368	S371	S373	S375	T376	R403	D405	R408	K417	N440	K444	V445	G446	N450	L452	L455	F456	N460	A475	S477	T478	N481	V483	E484	F486	F490	Q493	Q498	N501	Y505
D614G																																																																		
Delta		R												D					G	del	del																														R						K									
BA.2		I		del	del	del	S							D													G							D				F	P	F	A		N	S	N	K										N	K			A			R	R	Y	H
BA.2.75		I		del	del	del	S							D			E	R		L				V			G					S		S				F	P	F	A		N	S	N	K			S					K		N	K			A				R	Y	H
BA.2.86	ins MPLF	I	T	del	del	del	S	L		del	del		F	D	del					S	G				del	I	G	F	N	D			V	H		T		F	P	F	A	K	N	S	N	K		H	S	D	W			K		N	K	K	del	K	P			R	Y	H
JN.1	ins MPLF	I	T	del	del	del	S	L		del	del		F	D	del					S	G				del	I	G	F	N	D			V	H		T		F	P	F	A	K	N	S	N	K		H	S	D	W	S		K		N	K	K	del	K	P			R	Y	H
CH.1.1		I		del	del	del	S							D			E	R		L				V			G					S		H	T			F	P	F	A		N	S	N	K	T		S		R			K		N	K			A	S			R	Y	H
BA.5		I		del	del	del	S			del	del			D													G							D				F	P	F	A		N	S	N	K					R					N	K			A	V			R	Y	H
BQ.1.1		I		del	del	del	S			del	del			D													G							D	T			F	P	F	A		N	S	N	K	T				R			K		N	K			A	V			R	Y	H
FA.1		I		del	del	del	S			del	del			D	del												G							D	T			F	P	F	A		N	S	N	K	T				R			K		N	R			A	V			R	Y	H
XBB		I		del	del	del	S					A		D	del	Q							E				E							H	T		I	F	P	F	A		N	S	N	K		P	S					K		N	K			A	S	S		R	Y	H
XBB.1.5		I		del	del	del	S					A		D	del	Q							E				E				V			H	T		I	F	P	F	A		N	S	N	K		P	S					K		N	K			A	P	S		R	Y	H
EG.5		I		del	del	del	S					A		D	del	Q							E				E				V			H	T		I	F	P	F	A		N	S	N	K		P	S				L	K		N	K			A	P	S		R	Y	H
XBB.1.16		I		del	del	del	S					A		D	del	Q						V	E				E				V			H	T		I	F	P	F	A		N	S	N	K		P	S					K		N	R			A	P	S		R	Y	H
HV.1		I		del	del	del	S		H			A		D	del	Q				L			E				E				V			H	T		I	F	P	F	A		N	S	N	K		P	S		R		L	K		N	K			A	P	S		R	Y	H
HK.3		I		del	del	del	S		H			A		D	del	Q							E				E				V			H	T		I	F	P	F	A		N	S	N	K		P	S			F	L	K		N	K			A	P	S		R	Y	H
FL.15.1.1		I		del	del	del	S					A		D	del	Q							E				E				V			H	T		I	F	P	F	A		N	S	N	K		P	S			F	L	K	V	N	K			A	P	S		R	Y	H

Figure S5 | Key amino acid mutations in NTD and RBD in highlighted variants, related to Figure 5.

NTD, N-terminal domain. RBD, receptor binding domain.

Supplementary Tables

Table S1 | Properties of 20 fluorescence proteins involved in this study, related to Figure 1A.

Fluorescence proteins	Color group	Ex-Max (λ)	Em-Max (λ)	Fluorescence channels		Extinction coefficient (M^{-1} cm^{-1})	Quantum yield (%)	Oligomerization	Brightness	pKa	Molecular weight (kDa)
				Ex (λ)	Em (λ)						
mTagBFP2	Blue	399	454	390-420	430-500	50,600	64.0	Monomer	32.38	2.7	26.7
mTurquoise2	Cyan	434	474	435-460	470-515	30,000	93.0	Monomer	27.90	3.1	26.9
mTFP1	Cyan	462	492	435-460	470-515	64,000	85.0	Monomer	54.40	4.3	26.9
mGreenLantern	Green	503	514	460-490	515-580	101,800	72.0	Monomer	73.30	5.6	26.8
StayGold	Green	496	515	460-490	515-580	159,000	93.0	Dimer	147.87	4.0	24.6
mNeonGreen	Green/Yellow	506	517	460-490	515-580	116,000	80.0	Monomer	92.80	5.7	26.6
mAmetrine	Long Stokes Shift	406	526	390-420	500-550	45,000	58.0	Monomer	26.10	6.0	26.8
LSSmOrange	Long Stokes Shift	437	572	435-460	570-620	52,000	45.0	Monomer	23.40	5.7	26.7
CyOFP1	Long Stokes Shift	497	589	490-515	570-620	40,000	76.0	Monomer	30.40	5.5	26.4
mCRISPRed	Long Stokes Shift	460	592	435-460	570-620	28,500	46.0	Monomer	13.11	2.1	26.6
mRuby3	Red	558	592	530-560	570-620	128,000	45.0	Monomer	57.60	4.8	26.6
mScarlet-I	Red	569	593	530-560	570-620	104,000	54.0	Monomer	56.16	5.4	26.4
LSSmScarlet	Long Stokes Shift	470	598	460-490	570-620	30,200	42.0	Monomer	12.68	5.8	26.3
mBeRFP	Long Stokes Shift	446	611	435-460	570-620	65,000	27.0	Monomer	17.55	5.6	26.4
hmKeima8.5	Long Stokes Shift	438	612	435-460	570-620	32,000	34.0	Monomer	10.88	5.3	25.1
emiRFP670	Near-infrared	642	670	615-645	655-760	87,400	14.0	Monomer	12.24	4.5	34.2
iRFP670	Near-infrared	643	670	615-645	655-760	114,000	11.0	Dimer	12.54	4.0	34.5
miRFP670nano	Near-infrared	645	670	615-645	655-760	95,000	10.8	Monomer	10.26	3.7	17.1
miRFP713	Near-infrared	690	713	650-675	685-760	99,000	7.0	Monomer	6.93	3.5	34.6
emiRFP703	Near-infrared	674	703	650-675	685-760	90,900	8.6	Monomer	7.82	4.5	34.2

Ex, excitation wavelength. Em, emission wavelength. Data were obtained from FPbase (https://www.fpbase.org).

Table S2 | Human sera used in this study, related to the right panel of Figure 4A.

ID	Gender	Age	SARS-CoV-2 vaccination status	With past SARS-CoV-2 infection
S001	male	41	3-dose of IV	no
S002	male	26	2-dose of IV	no
S003	female	36	3-dose of IV	yes
S004	female	42	3-dose of IV	yes
S005	female	46	3-dose of IV	yes
S006	female	42	3-dose of IV	yes
S007	female	30	3-dose of IV	yes
S008	male	46	3-dose of IV	yes
S009	female	54	3-dose of IV	yes
S010	female	37	3-dose of IV	yes
S011	female	27	3-dose of IV	yes
S012	female	32	3-dose of IV	yes
S013	female	39	3-dose of IV	yes
S014	female	30	3-dose of IV	yes
S015	male	45	3-dose of IV	yes
S016	female	41	3-dose of IV	yes
S017	female	38	3-dose of IV	yes

IV, inactivated vaccine.

Table S4 | Detailed neutralizing antibody response profiles of immunized hamsters' sera, related to Figure 5B.

	BA.5			BQ.1.1			CH.1.1			XBB.1.5		
	GMT	rNT	P value	GMT	rNT	*P* value	GMT	rNT	*P* value	GMT	rNT	*P* value
**BA.5**	26164	-	-	22948	0.863	>0.9999	10277	0.933	>0.9999	6492	0.601	>0.9999
**DY.1.1**	27885	1.066	>0.9999	17580	0.661	>0.9999	5630	0.511	>0.9999	4580	0.424	>0.9999
**BF.7**	22960	0.878	>0.9999	21502	0.809	>0.9999	7764	0.705	>0.9999	6461	0.599	>0.9999
**BQ.1.1**	19913	0.761	>0.9999	26583	-	-	7325	0.665	>0.9999	3869	0.358	0.8470
**BQ.1.1.35**	12273	0.469	>0.9999	14617	0.550	>0.9999	5176	0.470	>0.9999	3841	0.356	0.8866
**BQ.1.1.71**	11827	0.452	>0.9999	13601	0.512	>0.9999	4872	0.442	>0.9999	6690	0.620	>0.9999
**ER.1.1**	10298	0.394	>0.9999	12286	0.462	>0.9999	3641	0.331	>0.9999	2852	0.264	0.1044
**EY.1**	9115	0.348	>0.9999	11998	0.451	>0.9999	3643	0.331	>0.9999	3384	0.314	0.2241
**FA.1**	5376	0.205	>0.9999	6005	0.226	>0.9999	2959	0.269	>0.9999	4485	0.415	>0.9999
**CH.1.1**	2112	0.081	0.0038	3170	0.119	0.0181	11011	-	-	4022	0.373	>0.9999
**DV.1.1**	3056	0.117	0.1751	3454	0.130	0.0841	7762	0.705	>0.9999	3744	0.347	>0.9999
**FK.1**	2771	0.106	0.1079	4412	0.166	0.8957	7321	0.665	>0.9999	4263	0.395	>0.9999
**CH.1.1.6**	2281	0.087	0.0095	3024	0.114	0.0337	10142	0.921	>0.9999	4978	0.461	>0.9999
**CH.1.1.16**	2172	0.083	0.0070	3609	0.136	0.0789	5666	0.515	>0.9999	3975	0.368	>0.9999
**CH.1.1.2**	1541	0.059	<0.0001	2420	0.091	0.0008	7477	0.679	>0.9999	2541	0.235	0.0385
**XBB**	1705	0.065	0.0003	2562	0.096	0.0373	9481	0.861	>0.9999	4719	0.437	0.8091
**XBB.2.3.2**	3596	0.137	>0.9999	3851	0.145	>0.9999	3938	0.358	>0.9999	7381	0.684	>0.9999
**XBB.1.28**	3184	0.122	>0.9999	3277	0.123	0.9411	10601	0.963	>0.9999	7113	0.659	>0.9999
**XBB.1.5.27**	2583	0.099	0.4104	3162	0.119	0.7324	3418	0.310	>0.9999	7653	0.709	>0.9999
**XBB.1.16**	2436	0.093	0.3886	2451	0.092	0.0385	7036	0.639	>0.9999	9606	0.890	>0.9999
**XBB.1.12**	2423	0.093	0.2088	3603	0.136	>0.9999	12797	1.162	>0.9999	10377	0.961	>0.9999

	BA.5			BQ.1.1			CH.1.1			XBB.1.5		
	**GMT**	**rNT**	**P value**	**GMT**	**rNT**	***P* value**	**GMT**	**rNT**	***P* value**	**GMT**	**rNT**	***P* value**
**FZ.1**	2339	0.089	0.1969	2246	0.084	0.0082	5063	0.460	>0.9999	5905	0.547	>0.9999
**XBB.1.5**	2335	0.089	0.1969	2216	0.083	0.0038	7230	0.657	>0.9999	10795	-	-
**XBB.1.5.30**	2307	0.088	0.0572	2297	0.086	0.0102	4831	0.439	>0.9999	7793	0.722	>0.9999
**XBB.1.5.3**	2292	0.088	0.1220	2726	0.103	0.1857	3446	0.313	>0.9999	5906	0.547	>0.9999
**XBB.1.5.12**	2178	0.083	0.0536	2465	0.093	0.0455	6302	0.572	>0.9999	8473	0.785	>0.9999
**XBB.1.5.1**	2155	0.082	0.0412	2361	0.089	0.0223	4826	0.438	>0.9999	5868	0.544	>0.9999
**FD.3**	1804	0.069	0.0020	2067	0.078	0.001	4477	0.407	>0.9999	5739	0.532	>0.9999
**XBB.1.17.1**	1768	0.068	0.0014	2254	0.085	0.0065	3534	0.321	>0.9999	6808	0.631	>0.9999
**XBB.1.5.2**	1728	0.066	0.0008	2295	0.086	0.0157	4689	0.426	>0.9999	5410	0.501	>0.9999
**EG.5**	1705	0.065	0.0038	1818	0.068	<0.0001	4655	0.423	>0.9999	7042	0.652	>0.9999
**EG.1**	1626	0.062	0.0002	1780	0.067	<0.0001	4250	0.386	>0.9999	6247	0.579	>0.9999
**XBB.1.5.4**	1588	0.061	0.0002	1571	0.059	<0.0001	3224	0.293	>0.9999	3889	0.360	0.1235
**EK.1**	1564	0.060	<0.0001	1763	0.066	<0.0001	3913	0.355	>0.9999	5564	0.515	>0.9999
**FG.1**	1484	0.057	0.0001	1689	0.064	<0.0001	4417	0.401	>0.9999	6493	0.602	>0.9999
**XBB.1.8**	1480	0.057	0.0002	2119	0.080	0.0011	5739	0.521	>0.9999	4935	0.457	0.7726
**XBB.1.36**	1312	0.050	<0.0001	1679	0.063	<0.0001	4166	0.378	>0.9999	3451	0.320	0.0586
**XBB.1.31**	1266	0.048	<0.0001	1551	0.058	<0.0001	3043	0.276	>0.9999	4498	0.417	0.4353
**HV.1**	842	0.032	0.0283	1349	0.051	0.3114	2717	0.290	>0.9999	7330	0.679	>0.9999
**HK.3**	707	0.027	0.0250	1076	0.040	>0.9999	12714	0.238	>0.9999	5683	0.526	>0.9999
**FL.15.1.1**	571	0.022	0.0220	921	0.035	0.0076	7530	0.218	>0.9999	5653	0.524	>0.9999
**BA.2**	8812	0.337	>0.9999	3510	0.132	0.3481	4352	0.247	>0.9999	2500	0.232	0.0170
**DS.1**	3523	0.135	>0.9999	3789	0.143	<0.0001	1595	1.155	0.0315	8945	0.829	>0.9999
**BA.2.75**	2151	0.082	0.0385	2221	0.084	<0.0001	1922	0.684	0.2483	6428	0.595	>0.9999
**XBF.7.1**	3462	0.132	>0.9999	3274	0.123	<0.0001	214	0.395	0.0017	8570	0.794	>0.9999

	BA.5			BQ.1.1			CH.1.1			XBB.1.5		
	**GMT**	**rNT**	**P value**	**GMT**	**rNT**	***P* value**	**GMT**	**rNT**	***P* value**	**GMT**	**rNT**	***P* value**
**XAY.1.1.1**	899	0.034	<0.0001	1353	0.051	<0.0001	144	0.145	0.0011	3435	0.318	0.0699
**JN.1**	730	0.028	0.0014	1385	0.052	<0.0001	1997	0.181	0.3222	5978	0.554	>0.9999
**BA.2.86**	512	0.020	0.0051	821	0.031	<0.0001	2072	0.188	0.5292	4593	0.425	>0.9999
**CM.8.1.1**	518	0.020	<0.0001	659	0.025	<0.0001	1922	0.175	0.2483	2493	0.231	0.0026
**D614G**	544	0.021	<0.0001	165	0.006	<0.0001	214	0.019	0.0017	158	0.015	<0.0001
**Delta**	391	0.015	<0.0001	119	0.004	<0.0001	144	0.013	0.0011	43	0.004	<0.0001

GMT, geometric mean titer. rNT, mean relative nAb titer. P-values were calculated using two-tailed Wilcoxon signed-rank tests of paired samples.
