# Supplementary material for: A quadri-fluorescence SARS-CoV-2 pseudovirus system for efficient antigenic characterization of multiple circulating variants
Source: Cell Rep Methods. 2024 Sep 6;4(9):100856. doi: 10.1016/j.crmeth.2024.100856 (PMC11440059; doi:10.1016/j.crmeth.2024.100856)
Supplement: Document S2. Article plus supplemental information [file mmc3.pdf]

Cell Reports Methods

Article

A quadri-fluorescence SARS-CoV-2 pseudovirus system for efficient antigenic characterization of multiple circulating variants

Graphical abstract

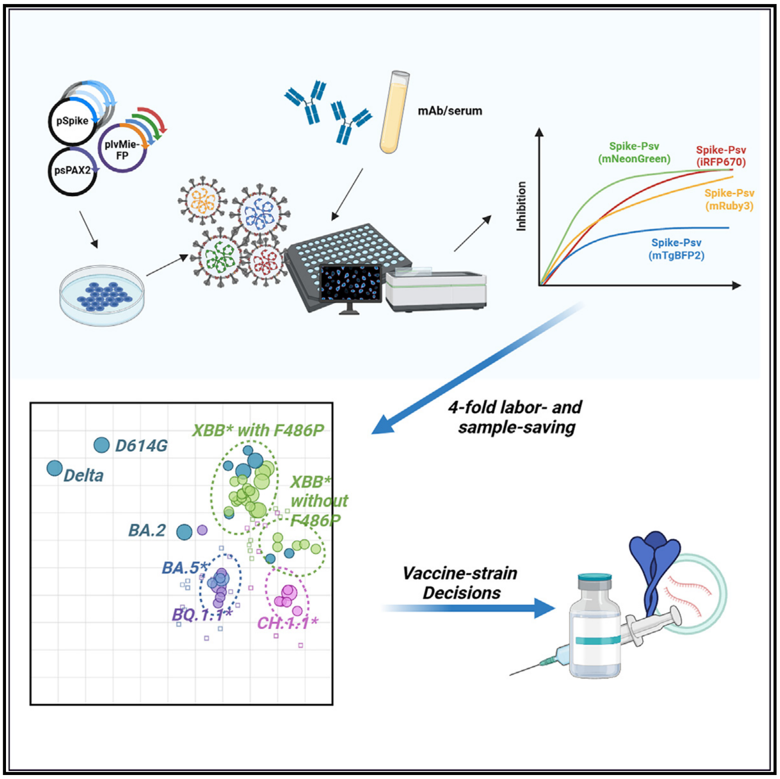

Highlights

- We develop a robust tool for high-throughput antigenic profiling of SARS-CoV-2
- qFluo is 4-fold labor and sample saving compared to traditional assays
- We show that the XBB.1.5 can still effectively induce a neutralizing response against JN.1
- The F486P results in antigenic differentiation within the progeny of the XBB

Authors

Jijing Chen (陈积璟), Zehong Huang (黄泽宏), Jin Xiao (肖瑾), ..., Ningshao Xia (夏宁邵), Quan Yuan (袁权), Tong Cheng (程通)

Correspondence

yangtaowu@xmu.edu.cn (Y.W.),
zhangyali@xmu.edu.cn (Y.Z.),
yuanquan@xmu.edu.cn (Q.Y.),
tcheng@xmu.edu.cn (T.C.)

In brief

Chen et al. develop a quadri-fluorescence pseudovirus platform using four fluorescent reporters with distinct spectra, enabling the simultaneous measurement of neutralizing antibodies against four SARS-CoV-2 variants in a single test. This method is conducive to informing vaccine strain decisions and understanding the evolutionary trajectory of SARS-CoV-2.

Chen et al., 2024, Cell Reports Methods *4*, 100856
September 16, 2024 © 2024 The Author(s). Published by Elsevier Inc.
https://doi.org/10.1016/j.crmeth.2024.100856

CellPress

Cell Reports Methods

CellPress
OPEN ACCESS

Article

A quadri-fluorescence SARS-CoV-2 pseudovirus system for efficient antigenic characterization of multiple circulating variants

Jijing Chen (陈积璟),[1,2,4] Zehong Huang (黄泽宏),[1,2,4] Jin Xiao (肖瑾),[1,2,4] Shuangling Du (杜双伶),[1,2,4] Qingfang Bu (布庆芳),[1,2] Huilin Guo (郭慧琳),[1,2] Jianghui Ye (叶江辉),[1,2] Shiqi Chen (陈诗琦),[3] Jiahua Gao (高佳华),[1,2] Zonglin Li (李宗霖),[1,2] Miaolin Lan (蓝妙琳),[1,2] Shaojuan Wang (王邵娟),[1,2] Tianying Zhang (张天英),[1,2] Jiming Zhang (张继明),[3] Yangtao Wu (巫洋涛),[1,2,*] Yali Zhang (张雅丽),[1,2,*] Ningshao Xia (夏宁邵),[1,2] Quan Yuan (袁权),[1,2,*] and Tong Cheng (程通)[1,2,5,*]

[1]State Key Laboratory of Vaccines for Infectious Diseases, Xiang An Biomedicine Laboratory, School of Public Health, School of Life Sciences, Xiamen University, Xiamen 361102, P.R. China
[2]National Institute of Diagnostics and Vaccine Development in Infectious Diseases, Collaborative Innovation Center of Biologic Products, National Innovation Platform for Industry-Education Integration in Vaccine Research, Xiamen University, Xiamen 361102, P.R. China
[3]Department of Infectious Diseases, Shanghai Key Laboratory of Infectious Diseases and Biosafety Emergency Response, Shanghai Institute of Infectious Diseases and Biosecurity, National Medical Center for Infectious Diseases, Huashan Hospital, Fudan University, Shanghai 200040, P.R. China
[4]These authors contributed equally
[5]Lead contact
*Correspondence: yangtaowu@xmu.edu.cn (Y.W.), zhangyali@xmu.edu.cn (Y.Z.), yuanquan@xmu.edu.cn (Q.Y.), tcheng@xmu.edu.cn (T.C.)
https://doi.org/10.1016/j.crmeth.2024.100856

MOTIVATION In the current context where multiple severe acute respiratory syndrome coronavirus 2 (SARS-CoV-2) variants are co-circulating, traditional monovalent neutralization assays undoubtedly present challenges to the ongoing development of COVID-19 vaccines and virological research on SARS-CoV-2. Here, we engineered a quadri-fluorescence (qFluo) lentiviral pseudovirus system employing four spectrally distinct fluorescent protein reporters, enabling the concurrent measurement of neutralizing antibodies (nAbs) against four SARS-CoV-2 variants within a single assay.

SUMMARY

The ongoing co-circulation of multiple severe acute respiratory syndrome coronavirus 2 (SARS-CoV-2) strains necessitates advanced methods such as high-throughput multiplex pseudovirus systems for evaluating immune responses to different variants, crucial for developing updated vaccines and neutralizing antibodies (nAbs). We have developed a quadri-fluorescence (qFluo) pseudovirus platform by four fluorescent reporters with different spectra, allowing simultaneous measurement of the nAbs against four variants in a single test. qFluo shows high concordance with the classical single-reporter assay when testing monoclonal antibodies and human plasma. Utilizing qFluo, we assessed the immunogenicities of the spike of BA.5, BQ.1.1, XBB.1.5, and CH.1.1 in hamsters. An analysis of cross-neutralization against 51 variants demonstrated superior protective immunity from XBB.1.5, especially against prevalent strains such as “FLip” and JN.1, compared to BA.5. Our finding partially fills the knowledge gap concerning the immunogenic efficacy of the XBB.1.5 vaccine against current dominant variants, being instrumental in vaccine-strain decisions and insight into the evolutionary path of SARS-CoV-2.

INTRODUCTION

Since the emergence of severe acute respiratory syndrome coronavirus 2 (SARS-CoV-2) in humans in late 2019, the virus has caused about 7 million deaths in the world.[1] Although the World Health Origination (WHO) has declared the end of the coronavirus 2019 (COVID-19) pandemic, the health influence caused by the SARS-CoV-2 infection continues worldwide. However, with successive COVID-19 epidemics in the next stage, multiple variants with varying spike antigenicity keep emerging. Emerging SARS-CoV-2 variants gain competitive edges through genetic drift toward continuously improving transmission fitness to propagate their offspring populations. In the current era of herd immunity, preexisting host immunity acquired from previous

This is an open access article under the CC BY license (http://creativecommons.org/licenses/by/4.0/).

infections or vaccinations has become the primary selection factor in directing virus evolution. Variants with more robust immune evasion capabilities are more likely to gain advantages in transmissibility. For example, the Omicron variants that emerged at the end of 2021 significantly evade the neutralizing antibodies (nAbs) elicited by the antigens of ancestral virus or the early variants of concern (VOCs), like Alpha, Beta, and Delta. The currently prevalent XBB and its numerous sub-lineages have further gained the capability to evade nAbs raised by early antigens of Omicron variants such as BA.1, BA.2, and BA.4/5.[2–6] Nevertheless, the co-existence of various evolving variants poses a significant challenge to selecting vaccine immunogens. Effective guidance in developing vaccine immunogens can only be achieved through timely analysis of the antigenic characteristics of prevalent variants.

SARS-CoV-2 pseudovirus (S2CoV-PsV) assays are a convenient and well-documented tool to determine the nAb titers raised by COVID-19 vaccinations or natural infections and are also helpful in evaluating the potencies of therapeutic or prophylactic monoclonal antibodies (mAbs). Compared with authentic virus tests, S2CoV-PsV assays based on lentiviral (LV) or vesicular stomatitis virus (VSV) vectors have advantages in efficiency, availability, and biosafety, thereby being widely used.[7–10] The nAb titers determined by LV S2CoV-PsV neutralization tests have been demonstrated to be correlated with protection efficacies in the COVE and ENSEMBLE COVID-19 vaccine phase 3 clinical trials.[11,12] In addition, cross-variant neutralization using spike variants bearing S2CoV-PsVs can provide essential information for mapping antigenic relationships of multiple SARS-CoV-2 lineages and sub-lineages.[13–15] However, most previously described S2CoV-PsV systems based on fluorescent protein or luciferase reporters can only support single-channel tests per sample against each virus, which is time consuming and labor intensive in cross-neutralization assessments against multiple variants, especially in the current stage that various co-existing variants exhibit diverse antigenicity. In this study, we established a quadri-fluorescence (qFluo) LV PsV system using four fluorescent protein reporters (mTagBFP2, mNeonGreen, mRuby3, and iRFP670) with different spectra, which allows simultaneous neutralization assessments for a blood or mAb sample against 4 SARS-CoV-2 variants in a single test. Compared with the classical mono-fluorescence (mFluo) assay, we demonstrated that qFluo presented highly consistent neutralization results with 4-fold labor and sample saving in detecting various samples, including human plasmas, mAbs and animal immunized sera. The qFluo assay provided a high-throughput tool for antigenicity characterizations of circulating SARS-CoV-2 spike variants, and it can also be adapted to develop multichannel infection reporting systems for other viruses.

RESULTS

Constructions and evaluations of multichannel FP reporters for S2CoV-PsV

The green fluorescence proteins (GFPs) are the most commonly used reporter for S2CoV-PsV infection visualization. Besides GFPs, numerous fluorescence proteins (FPs) with various spectra profiles have been discovered. A set of FP combinations with minimal spectral overlap and low spillover spreading is essential to develop multicolor S2CoV-PsV reporters. We first constructed 20 FPs with excellent cellular brightness into the LV shuttle vector (pLVEF1α). The properties of 20 FPs[16–33] involved in our study are listed in Table S1. For the pLVEF1α-FPs vectors, the expressions of FPs are driven by a human EF1α promoter. In transient transfection tests in 293T/17 cells, all exhibited detectable fluorescence in the corresponding excitation/emission channels (Figures 1A and S1). Considering the cellular brightness and absence of spillover spreading, we selected mTagBFP2, mNeonGreen, mRuby3, and iRFP670 as the combination set for multicolor reporters (Figure 1A, right). Co-transfections of the pLVEF1α-FP, psPAX2, and SARS-CoV-2 spike expression plasmids in 293T/F17 cells yielded infectious S2CoV-PsVs, which successfully showed the expected fluorescence signals without spillover spreading during infection on H1299-hACE2 cells (Figure 1B). In addition, we found that a modified shuttle vector (pLVMie) with an hCMVmie promoter-driven FP expression cassette exhibited significantly brighter fluorescence than pLVEF1α-FP when used in S2CoV-PsVs (Figure 1B). Therefore, we used the improved pLVMie-FP plasmids to produce S2CoV-PsVs for subsequent studies. As the workflow illustrates in Figure 1C, we established the qFluo assay, which simultaneously utilized four different spike-variant-bearing S2CoV-PsVs with four different FP reporters to infect the H1299-hACE2 cells. The infected cells by different S2CoV-PsV variants can be calculated using a fully automatic high-content imaging system at the corresponding channels (Figure 1C). Other steps followed a process similar to the traditional pseudotyped virus neutralization assay.[7,9]

Establishments and validations of the S2CoV-PsV qFluo neutralization assay

When cells are co-infected with multiplex variants, a single cell may be simultaneously infected by two or more viruses. We tested the dose-dependent manner between the co-infection and virus dosage. As shown in Figure 2, with the increase in infection dosage, the number and proportion of cells displaying two or more fluorescence signals rose significantly, indicating that most cells were simultaneously infected by two or more viruses under high dosage conditions (Figure 2A). We confirmed the visual observation by employing uniform manifold approximation and projection (UMAP) to cluster reporter expression levels (Figure 2B). For example, at a dosage of 18,000 fluorescence-forming units (FFU)/well, over 80% of cells were simultaneously infected by four viruses (Figure 2C). In contrast, the number and proportion of positive cells infected by a single virus noticeably increase under relatively low virus dosage conditions. At a 1,125 FFU/well dosage, approximately 67% of infected cells only exhibited a single fluorescence (Figure 2C). Next, we use the recombinant ACE2 protein (rhuACE2) as a broadly neutralizing antibody surrogate to test the qFluo performance in neutralization tests. We measured the potencies (half-maximal inhibitory concentration, IC_{50}) of rhuACE2 in neutralizing S2CoV-PsV qFluo variants of D614G, Beta, Delta, and BA.1 at various viral dosages. When the S2CoV-PsV used ranges from 1,000 to 2,500 FFU/well, the rhuACE2 showed relatively constant IC_{50} values against these variants (Figure 3A). More importantly, the qFluo assay showed

CellPress
OPEN ACCESS

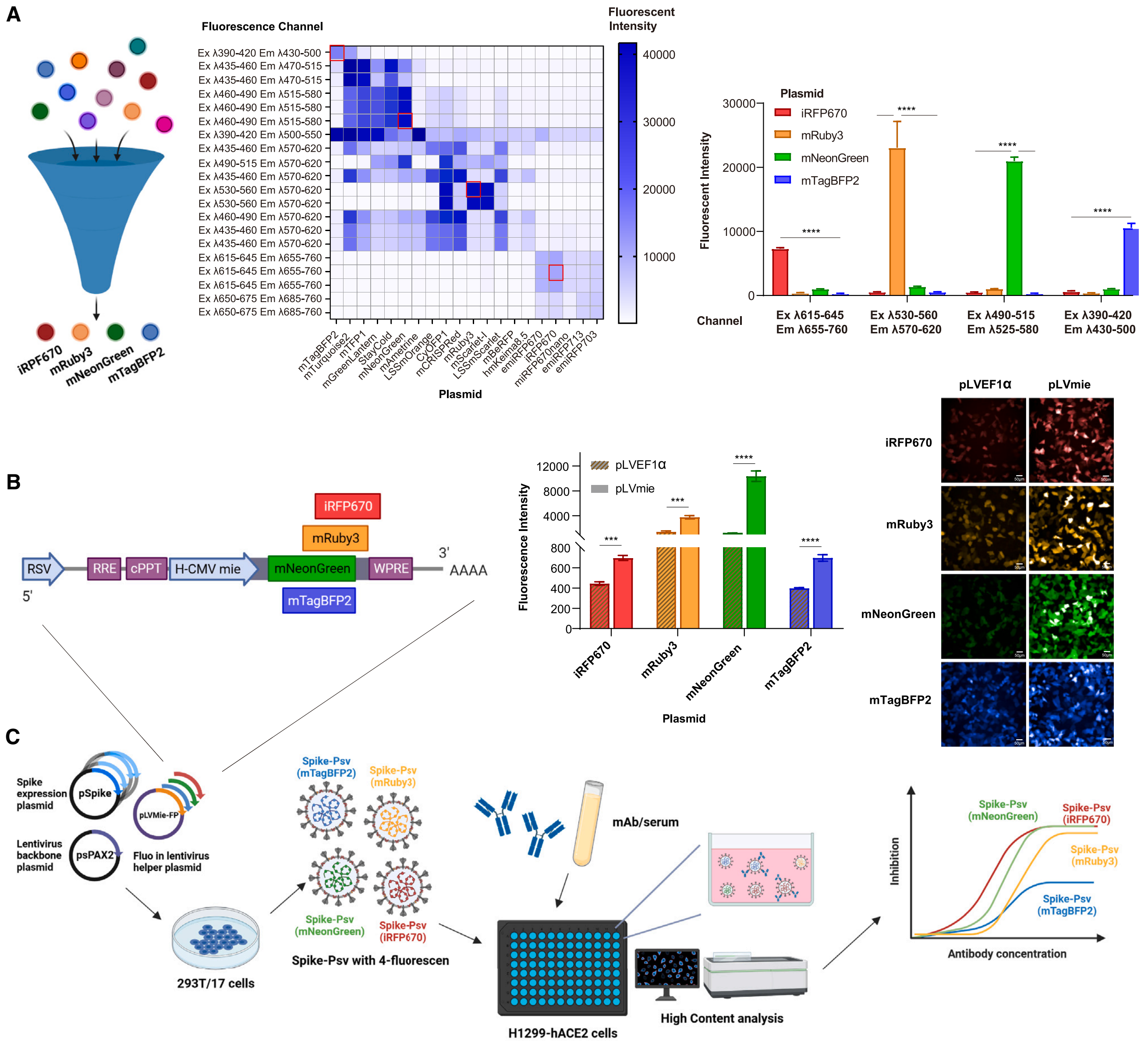

Figure 1. The design and operation flow of the qFluo neutralization assay

(A) Screening process of four fluorescent proteins. The middle and right images depict the fluorescence intensity of 20 fluorescent proteins used for screening in different fluorescence channels, as well as the intensity performance of the four selected fluorescent proteins in their respective fluorescence channels, respectively. Results are from fluorescence field-of-view 293T/17 cells transfected with each fluorescent plasmid for 48 h.

(B) A significant elevation in fluorescence expression intensity was noted post the substitution of the promoter driving the reporter gene. The schematic representation of the pLV.Fluo plasmid is illustrated on the left, while the middle and right images depict quantified and unquantified fluorescence expression outcomes of H1299-hACE2 cells infected with each pseudotyped lentivirus for 48 h after promoter replacement, respectively.

(C) Approach for operation qFluo neutralization assay. Combine various spike expression plasmids with lentiviral helper plasmids (PlvMie-FP) carrying different fluorescent genes, along with lentiviral backbone plasmids (psPAX2), and co-transfect them into 293T/17 cells to package pseudotyped virus with the fluorescence corresponding to spike specifically. Subsequently, the four pseudoviruses (PsVs) were co-incubated with monoclonal antibody (mAb) or serum in H1299-hACE2 cells. Inhibition rates were calculated using high content analysis to assess the neutralization against different spike PsVs (spike-PsVs).

Data on the right of (A) and in the middle of (B) were plotted as the mean with SD. Dunnett's multiple comparison test and t test comparison were used for statistical comparisons. ****$p < 0.0001$ and ***$p < 0.001$. RRE, Rev response element; cPPT, central polypurine tract; WRPE, woodchuck hepatitis virus post-transcriptional regulatory element.

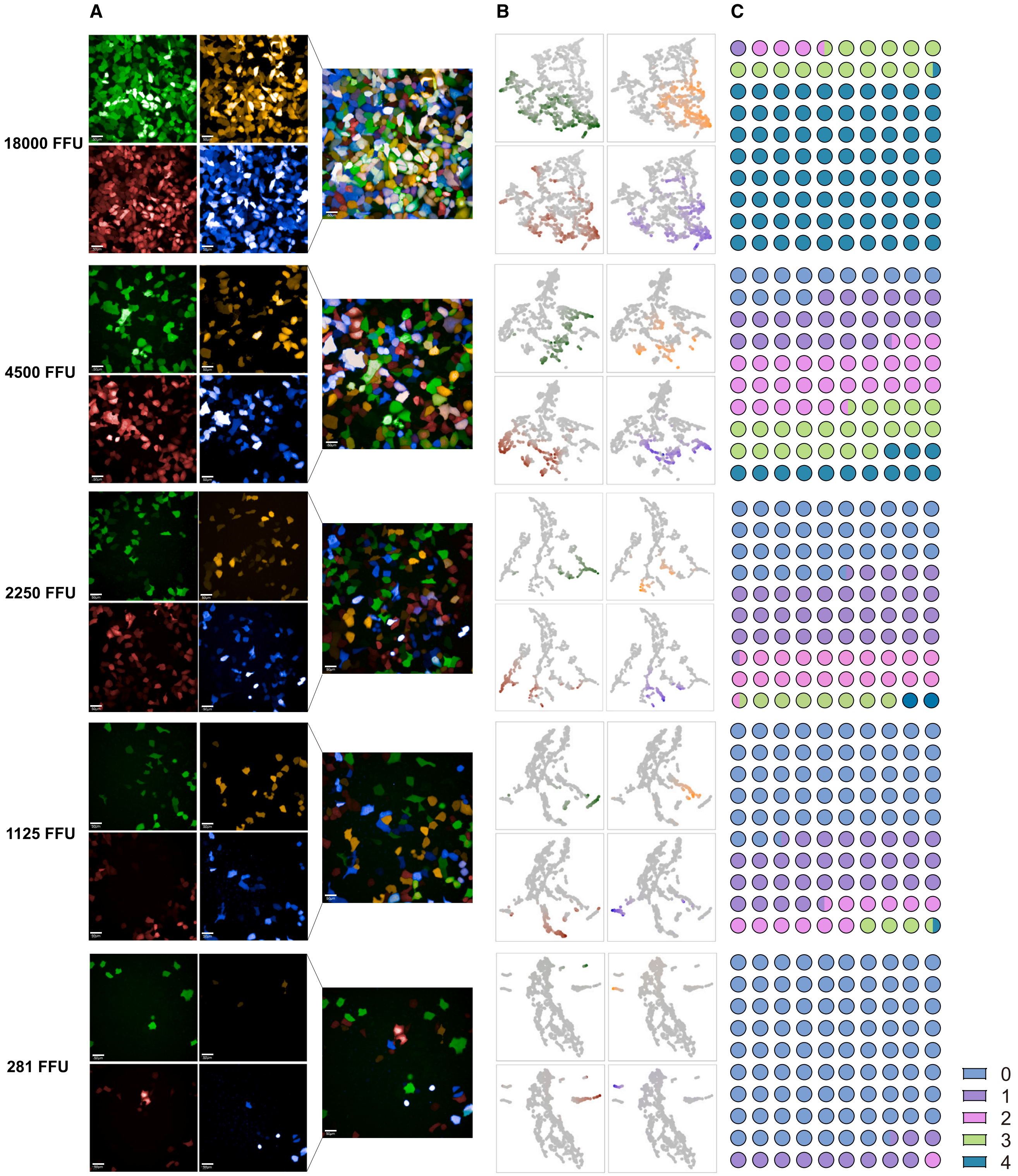

Figure 2. Excessively high infection dosage results in concurrent infection of multiple viruses within an individual cell

(A) The infection status of different fluorescence channels under various infection dosages. A four-panel diagram and the integrated pattern on the right depict the infection status of individual cellular pores across distinct fluorescence channels.

(legend continued on next page)

highly consistent IC_{50} and neutralizing curves with those from the classical mFluo reporter assay for all tested variants under an optimal infection dosage (1,800 FFU/well) (Figure 3B).

To further validate the qFluo assay, we selected 11 SARS-CoV-2 neutralizing mAbs targeting various epitopes of the spike protein[6,34–41] and 14 human plasma samples collected from vaccinated individuals or people who recovered from past SARS-CoV-2 infections (Table S2). These samples were subjected to both the qFluo and mFluo assays to assess their neutralization potencies (for mAbs) or nAb titers (for plasmas) against the D614G, Beta, Delta, and BA.1 spike variants, respectively. All mAbs and plasma samples exhibited similar dose-dependent infection-inhibitory curves for all four tested variants in the qFluo and mFluo systems (Figures S2 and S3). As expected, the potencies of mAbs (IC_{50}) and the nAb titers of plasmas (median infectious dose, ID_{50}), derived from the two systems, exhibited a strong positive correlation with correlation coefficients over 0.9 for each variant (Figure 4A). In reproducibility evaluations, we used the LY-CoV1404 mAb and three human plasma samples to test the coefficient of variation (CV) of the qFluo assay to determine the IC_{50} (for mAbs) and ID_{50} (for plasmas) values. In 3 independent batches of measurements for these samples (9 technical replicates for each sample), the average intra- and interassay CVs were estimated to be 12.3% and 15.9%, respectively (Figure 4B). These results demonstrated the quantitative accuracy of the S2CoV-PsV qFluo neutralization assay.

The qFluo provided a robust tool for antigenic profiling of multiple SARS-CoV-2 spike variants

In the present stage of co-circulation of multiple variants, conducting thorough immunological assessments on variants with distinct antigenic characteristics holds significant importance in enhancing our understanding of immune evasion potentials and the selection of vaccine antigens. Consequently, we employed the qFluo system to conduct neutralization assays on sera obtained from hamsters previously immunized with four representative spike variants antigens, namely BA.5, BQ.1.1, CH.1.1, and XBB.1.5 (Figure 5A). In this experiment, the four groups of hamsters were immunized with two doses of a recombinant spike protein subunit vaccine based on the abovementioned 4 variants. Immunized sera were collected at 2 weeks after a second dose to measure their nAb titers against 51 sub-lineages of SARS-CoV-2 variants using the S2CoV-PsV qFluo assay. The tested 51 SARS-CoV-2 sub-variants included 3 BA.5-related sub-lineages, 6 BQ.1.1-related sub-lineages, 6 CH.1.1-related sub-lineages, 25 XBB-related sub-lineages, and 11 other lineages, including BA.2.86 and JN.1 (Table S3). The geometric mean titer (GMT), individual reactivity profiles, and relative nAb changes for these sera are shown in Figure S4. All four tested recombinant protein immunogens were able to elicit high antibody levels (ID_{50}, GMT > 10,000) in neutralizing the corresponding S2CoV-PsV variants, thus demonstrating their strong immunogenicity.

As the results show in Figure 5B, it was observed that the forepassed D614G and Delta demonstrated significant escape tendencies across all sera, showing >50× nAb GMT reduction for all four groups of sera. Furthermore, antibodies elicited by the BA.5 and BQ.1.1 antigens only showed breadth in the neutralizing variants of sub-lineages of BA.5 and BQ.1.1 themselves. The mean relative nAb titers (rNTs) of sera of the two groups against the abovementioned variants were over 0.2 (<5× nAb GMT reduction; Figure S4; Table S4). However, the BA.5/BQ.1.1-immunized animal sera showed markedly decreased neutralization activities against XBB-related variants (6.9–45.8× nAb GMT reduction), CH.1.1-related variants (6.0–17.0× nAb GMT reduction), and several other variants, like DS.1, BA.2.75, XBF.7.1, XAY.1.1, CM.8.1.1, D614G, Delta, BA.2.86, and JN.1 (Figure S4). This undoubtedly suggests that the neutralizing protection afforded by booster vaccines using BA.5 as an antigen might experience a substantial decline against newly emerging variants in 2023 and beyond. As such, continued administration of booster vaccines appears almost inevitable. Interestingly, our findings indicate that BA.5 exhibits relatively broader cross-reactivity against earlier variants, such as BA.2, Delta, and D614G, compared to BQ.1.1. This could, to some extent, suggest that the R346T, K444T, and N460K mutations on the receptor-binding domain (RBD) of BQ.1.1 may significantly affect the antigenic characteristics in comparison to those elicited by previous strains (Figure S5).

Compared to BA.5 and BQ.1.1, the XBB.1.5 and CH.1.1 antigens induced nAbs with improved breadth to the variants tested in our study, including recently emerged variants such as HV.1, FL.15.1.1, HK.3, BA.2.86, and JN.1. The XBB.1.5- and CH.1.1-immunized sera exhibited high nAb titers and rNT levels to most variants except D614G and Delta (Figures 5B and S4; Table S4). It is encouraging to observe that XBB.1.5 has shown relatively good neutralization breadth against newly emerged variants EG.5, HV.1, FL.15.1.1, HK.3, BA.2.86, and JN.1, with rNTs all over 0.2 (<5× nAb GMT reduction; Figures 5B and S4; Table S4). These findings are in line with the trends seen in recent studies[42–45] and suggest that individuals vaccinated with an XBB.1.5-based vaccine or those who have had an infection with a virus sharing antigenic characteristics with XBB.1.5 in 2023 may still possess neutralizing protection against the currently dominant JN.1.

The antigenic cartographies based on data from the qFluo neutralization assays for these sera offer an alternative perspective for understanding the antigenicity differences of multiplex SARS-CoV-2 spike variants (Figures 5C and 5D). Two early lineages of D614G and Delta exhibited a significant antigenic disparity to other assessed sub-lineages of Omicron, with an antigenic distance greater than 6 antigenic units (AU), confirming the substantial antigenicity change of Omicron variants. The antigenic divergence between various sub-variants and the early Omicron BA.2 has progressively increased, with all antigenic

(B) The visualization of infection status using uniform manifold approximation and projection (UMAP). Perform UMAP dimensionality reduction and clustering on the four fluorescence intensities and colorize them using normalized fluorescence intensity values. Each quadrant represents the distribution range of cells with different fluorescence intensities. Darker colors indicate stronger fluorescence intensities, while gray represents weak fluorescence intensities.
(C) The proportion of cells simultaneously infected with different numbers (1, 2, 3, and 4) of viruses in the total cell population. FFU, fluorescence-forming unit.

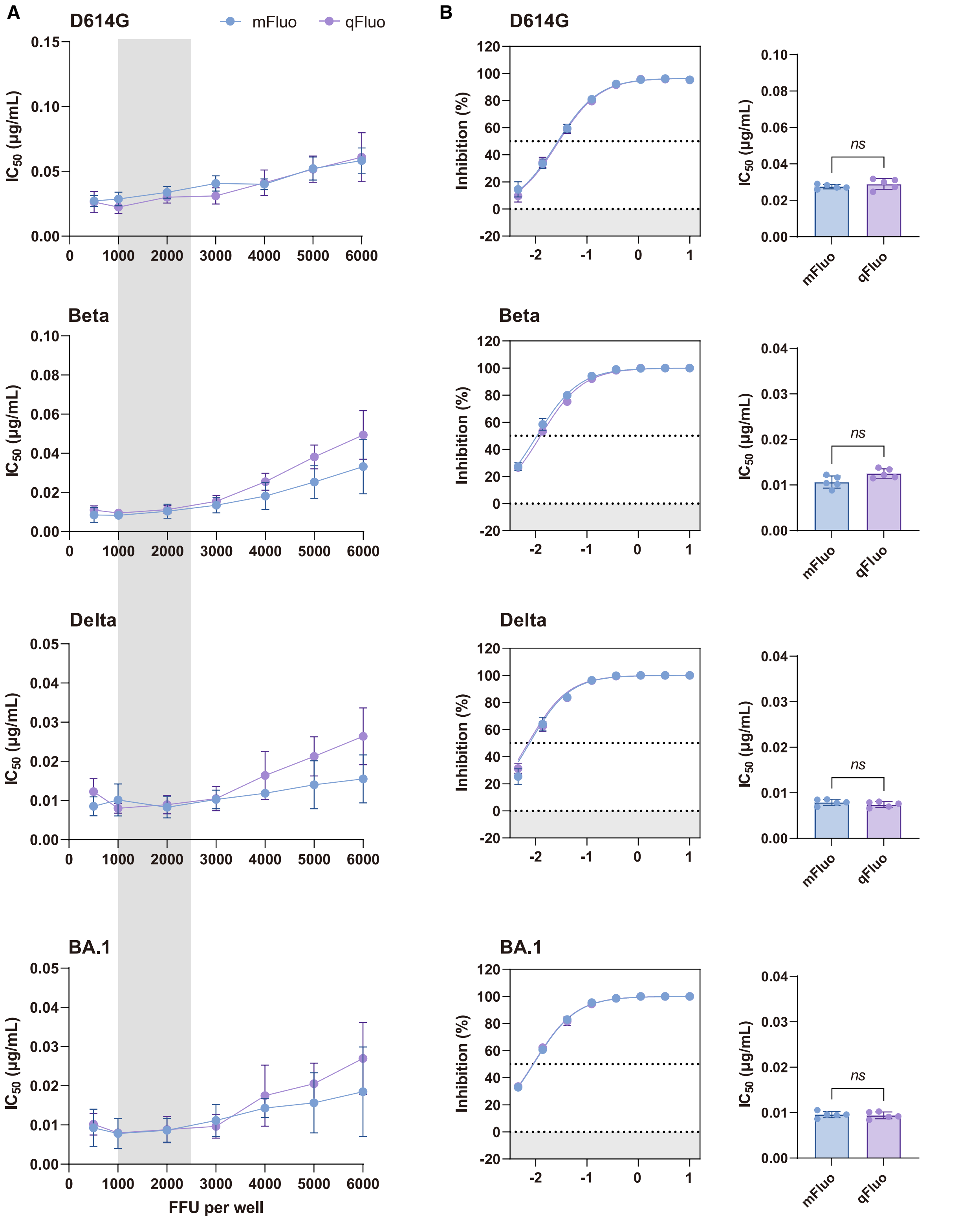

(legend on next page)

distances exceeding 2 AU, demonstrating a clear temporal evolutionary effect.

Additionally, the variants under study can generally be categorized into four clusters (Figure 5D). CH.1.1 and its sub-lineages constitute a distinct antigenic cluster. BA.5, BQ.1.1, and their sub-lineages form a single cluster, with distances mostly within 1 AU. An exception is FA.1, whose antigenic characteristic appears substantial compared to its ancestors, likely due to its distinctive mutations, Y144del and K478R. Meanwhile, the XBB lineage and its sub-lineages distinctly bifurcate into two clusters based on the presence of the F486P mutation. XBB.1.5 and XBB have an antigenic distance of 1.9 AU, comparable to that of BA.2.86. The F486P mutation notably accentuates the phylogenetic relationship between XBB sub-lineages and their BA.2.75 ancestor. Observations of lineages with L455F or F456L mutations reveal that the antigenic distance between XBB.1.5 and lineages such as EG.5 and HV.1 (0.6–1.1 AU), which only carry F456L, further expands when acquiring the L455F mutation, as seen with the "FLip" variants HK.3 and FL.15.1.1 (1.4–1.5 AU). Interestingly, JN.1, which carries L455S, narrows the distance to XBB.1.5 to 1.5 AU, compared to 1.8 AU for BA.2.86.

DISCUSSION

nAbs acquired from natural infections or vaccinations essentially contribute to the protection of SARS-CoV-2 infection and disease. In the current scenario of multiplex variant co-circulation, viral antigenic draft caused by spike amino acid substitutions is a dominant selection force for a virus to bypass herd immunity. Continuous antigenic characterization of new SARS-CoV-2 variants and a deep understanding of the viral evolutionary trajectory are key bases for developing the next generation of COVID-19 vaccines. Cross-variant neutralization profiling is the principal approach to assessing the antigenic diversity among circulating strains of SARS-CoV-2. Numerous studies have explored high-throughput approaches for neutralization experiments, aiming to enhance the efficiency of neutralization testing. Among reported methods, the cell-free surrogate virus neutralization test (sVNT), based on biochemical assays of the antibody inhibition effects on the RBD/ACE2 protein interaction, provided a high-throughput approach for SARS-CoV-2 nAb titrations.[46] Following the same principle, modified flow cytometry assays using RBD/spike protein-conjugated microparticles with different levels of fluorescence allow multiplexed measurements of nAbs against SARS-CoV-2 variants.[47,48] However, due to the absence of the virus, these methods similarly struggle to reflect the impact of viral entry processes on neutralization effects. These methods can only reflect the neutralizing effects of antibodies that directly inhibit the interaction between the SARS-CoV-2 RBD and ACE2, failing to adequately account for the neutralizing effects of antibodies that exert steric hindrance or fusion inhibition, such as non-ACE2-blocking RBD-targeting nAbs (like S309, CR3022, and hu33 mAbs)[49–51] and the N-terminal domain (NTD)- and S2-targeting nAbs.[37,52] This limitation may underestimate the neutralizing efficacy of test samples.[53] Therefore, the RBD/ACE2-binding-blocking-based sVNT assays do not substitute for infection-based neutralization assays. In the field of virus-cell-based neutralization assays, Benjamin et al. described a dual-reporter (GFP and mCherry) VSV-based SARS-CoV-2 PsV neutralization assay.[54,55] However, aside from its application in dengue,[56] multiplex PsV neutralization assays with over 2 channels have not been described.

Aiming to further improve the efficiency of antigenic profiling of SARS-CoV-2 spike variants, we established a high-throughput, multichannel PsV neutralization system (qFluo). This assay employs a spike-fluorescence matching approach to achieve a four-in-one infection mode. After screening from 20 reported FPs, we selected mTagBFP2, mNeonGreen, mRuby3, and iRFP670, which have excellent cellular brightness and non-interfering excitation-emission spectra, as qFluo reporters for S2CoV-PsV infections (Figure 1A). Notably, the four reporters could be measured by fluorescent microscopes or multimode microplate readers commonly used in most virological laboratories, ensuring this system's availability. By meticulously optimizing the vector and experimental parameters, we have significantly bolstered the robustness and reproducibility of this system. Extensive validation tests using mAbs and human plasmas demonstrated that the qFluo assay yielded neutralization data consistent with those derived from the traditional mono-reporter strategy (Figures 4A and 4B).

qFluo-based antigenic profiling, involving 51 spike variants, is consistent with findings described in previous investigations[4,5,57–59]; our data showed that the BA.5/BQ.1.1-immunized sera exhibited markedly reduced nAb activities to XBB.1.5, CH.1.1, and their descendants. Notably, our results revealed that antibodies acquired from immunization of XBB.1.5 spike have demonstrated a relatively broad neutralizing potency against the recently emerged "FLip" variants such as HK.3 and FL.15.1.1, which carry the F456L and L455F mutations, as well as against JN.1, a variant that has recently become predominant in multiple countries.[60] These findings indicate that existing vaccines utilizing XBB.1.5 as an antigen remain effective for neutralizing protection in the current epidemic. In contrast, individuals who have been neither vaccinated with this vaccine nor infected with a virus possessing similar antigenic characteristics as XBB.1.5 are at a significantly increased risk of infection. Given the drastic decline in global COVID-19 vaccination rates since late 2022,[61] the sustained promotion of COVID-19 vaccinations holds long-term and profound significance for the control of the epidemic.

Moreover, our observations indicate that the F486P substitution has induced differentiation in the antigenic characteristics within the XBB sub-lineages, thereby magnifying the antigenic

Figure 3. Determination of the optimal infection dosage range

The IC_{50} of rhuACE2 using the qFluo and mFluo neutralization systems under various infection dosages (A) and the optimal infection dosage range (1,800 FFU/well) (B). The number labeled in the top left corner of (A) represents the viral titer added to the first well. Data in (A) and on the left of (B) were plotted as the mean with SD on the right of (B) were plotted as the geometric mean and geometric SD. Mann-Whitney U test was used for intergroup statistical comparisons. FFU, fluorescence-forming unit; ns, not significant.

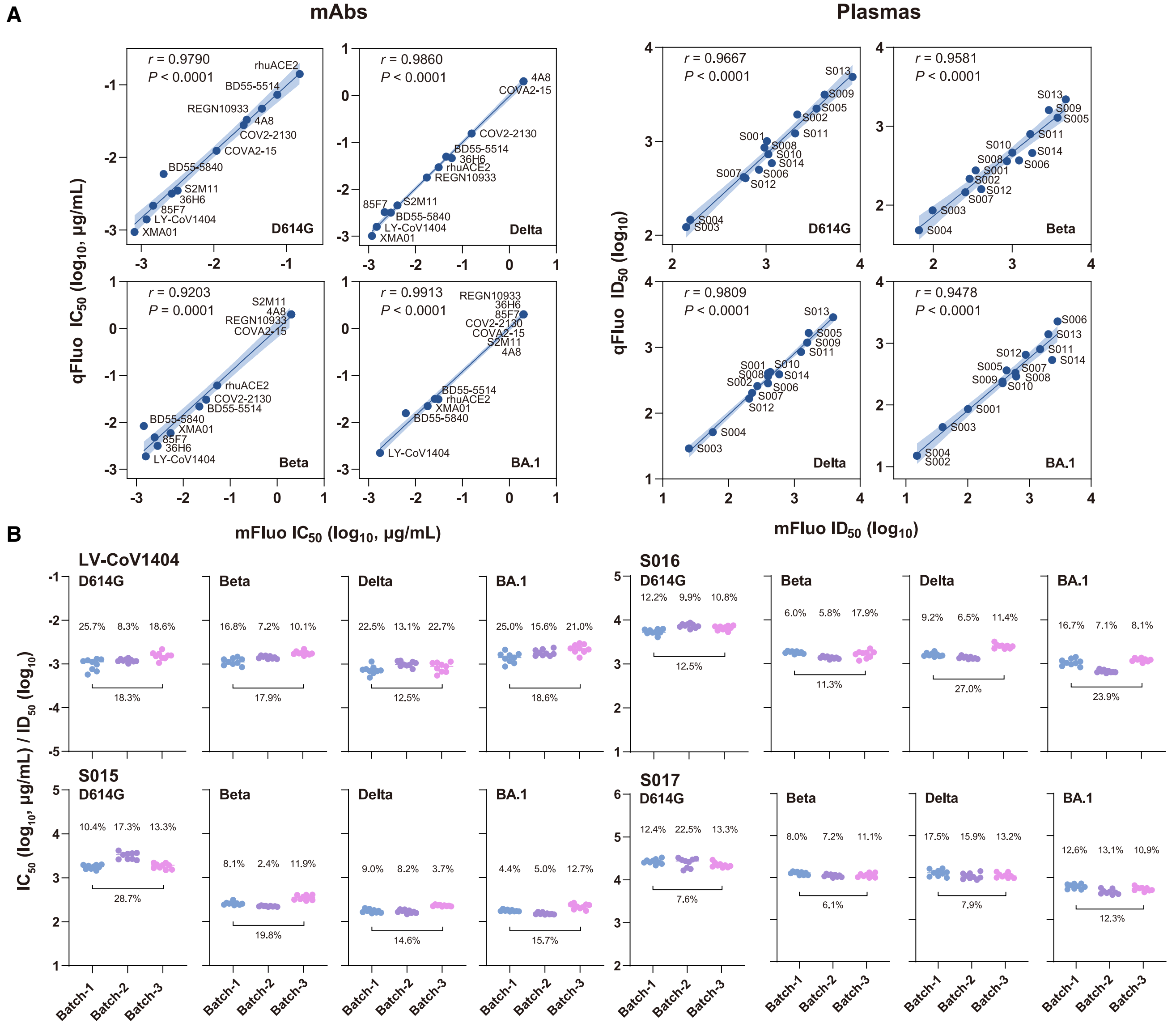

Figure 4. Validation and reproducibility of the qFluo neutralization assay

(A) Correlation of mFluo and qFluo neutralization assays to mAbs and plasmas test results. Data were plotted as IC_{50} or ID_{50}. p values resulted from a two-tailed test for the Spearman rank correlation coefficient.

(B) Reproducibility results of qFluo. The tests were repeated on three batches at different times, and 9 replicates of the samples were tested in each batch. The percentage above the scatter points is the intra-assay coefficient of variation (CV), and the one below is the interassay CV. CV is defined as the ratio of the SD to the mean. The average intra- and interassay CVs were estimated as 12.3% and 15.9%, respectively.

distance with ancestral BA.2.75 to a certain extent. Another interesting finding from our qFluo-based antigenic profiling was that some newly emerged variants, such as FLip and JN.1, did not exhibit markedly enhanced nAb escape capability, particularly to sera immunized by XBB.1.5. From an evolutionary perspective, this perhaps signifies that the antibodies induced by XBB.1.5 and its descendant have not exerted sufficient selective pressure on the current viral evolution. Evasion of the immunity acquired from infections caused by past variants like BA.5 or BQ.1.1 and fitness improvements on other virological aspects may still be the dominant force to drive the evolution of XBB sub-lineages. Although the potential of hamster sera antigen characterization has been validated,[62] these findings resulted from animals exposed to a single SARS-CoV-2 antigen, which may not fully represent humans. Antibody profiles of vaccinated humans with breakthrough SARS-CoV-2 infection one or more times are complicated and warrant further investigations.

In summary, we developed a quadri-color SARS-CoV-2 PsV system, qFluo, which provides a robust tool for high-throughput cross-variant neutralization assay for mAbs or polyclonal antibodies. The qFluo assay exhibited highly consistent results with the traditional single-channel method in assessments of

CellPress
OPEN ACCESS

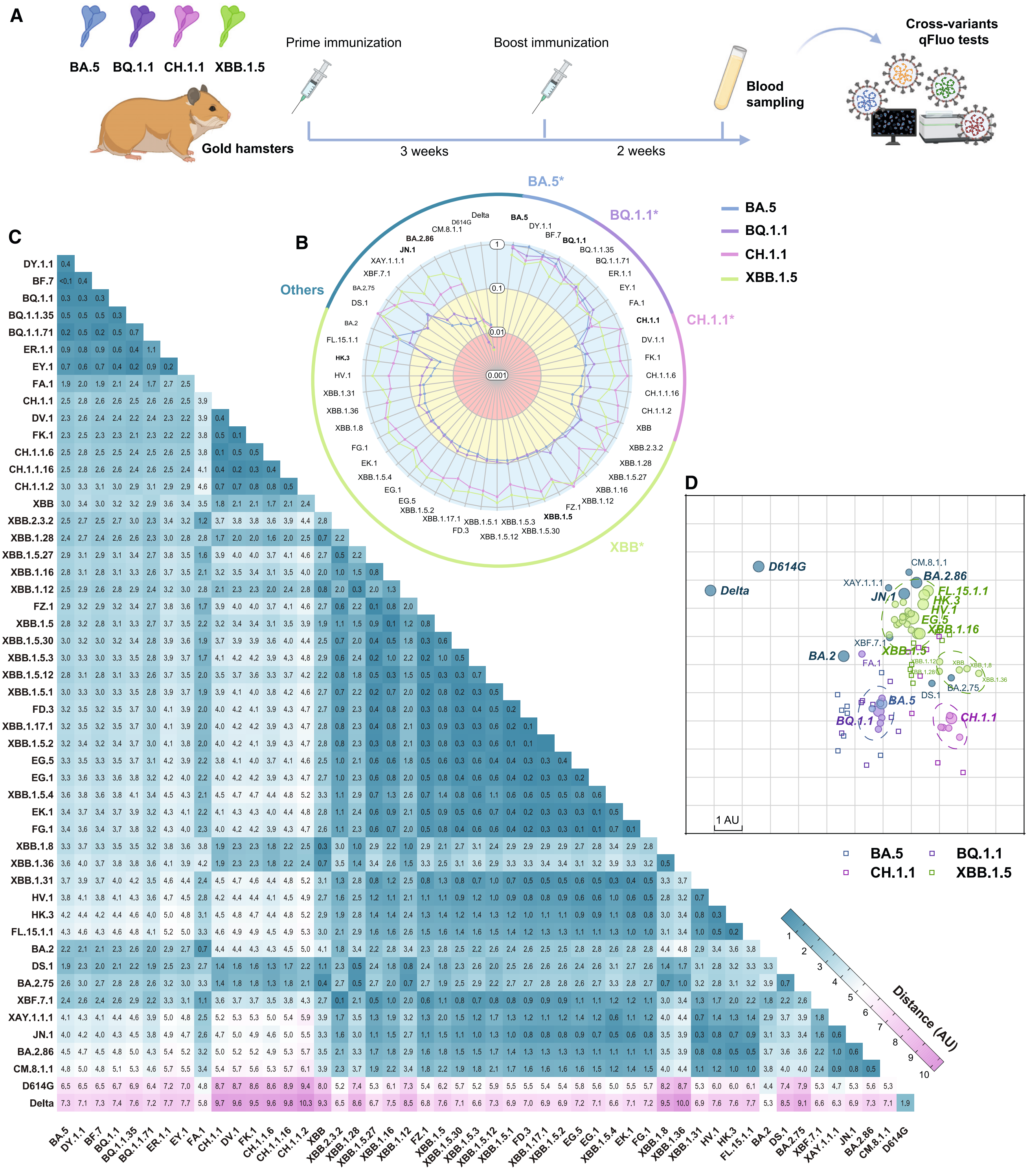

Figure 5. The qFluo neutralization assay for antigen escape ability evaluation

(A) Schematic time course of animal experiment.

(B) The radar chart is based on rNTs against various variants induced by different antigens.

(C and D) The antigenic distances between each variant (C) and antigenic cartography (D) based on NTs of all 32 sera against various variants induced by different antigens. Both axes represent antigenic distance with one antigenic distance unit (AU) in any direction corresponding to a 2-fold change in the neutralization ID_{50} titer. The circle represents the variant and the square represents the serum in the antigenic cartography.

anti-SARS-CoV-2 neutralization activities for samples of various types. Due to its four-in-one feature, the qFluo tool is 4-fold labor and sample saving, thereby enabling efficient antigenic profiling for multiple SARS-CoV-2 variants. Utilizing the qFluo tool, we demonstrated that the immunity elicited by XBB.1.5 remains effective in providing protection against recently dominant variants, such as FLip and JN.1, thereby partially addressing the gap in knowledge regarding the XBB.1.5 vaccine's immunogenic power against prevalent variants. Further applications of the qFluo tool will facilitate studies on SARS-CoV-2 antigenic evolution and the development of next-generation COVID-19 vaccines.

Limitations of the study

While qFluo is labor and sample saving, it requires multicolor imaging equipment. Moreover, this method employs a pseudotyped virus system, which may differ from live-virus-based assays in some cases.

RESOURCE AVAILABILITY

Lead contact

Further information and requests for resources and reagents should be directed to and will be fulfilled by the lead contact, Tong Cheng (tcheng@xmu.edu.cn).

Materials availability

All reagents, which include antibodies, proteins, plasmids, and viruses, will be made available for non-commercial usage upon request to the lead contact author after the completion of a materials transfer agreement.

Data and code availability

- The properties of FPs involved in this study are available from the corresponding references and FPbase (https://www.fpbase.org).
- This paper does not report any original code.
- Additional information required to reanalyze the data reported in this paper is available from the lead contact upon request.

ACKNOWLEDGMENTS

This study was supported by the National Natural Science Foundation of China: 92369110 (to Q.Y.), 82272305 (to Y.Z.), and 82272310 (to T.C.). The graphical abstract and some figure components in Figures 1 and 5A were created with BioRender.com.

AUTHOR CONTRIBUTIONS

Z.H., J.C., J.Z., Y.W., Y.Z., N.S., T.C., and Q.Y. conceptualized and designed the study. Z.H., J.X., Q.B., J.G., Z.L., and Y.W. designed the clones and produced and characterized the proteins. J.G. and Y.W. performed the animal experiments. S.C. and J.Z. enrolled the patients and collected the samples. J.C., Z.H., S.D., H.G, J.Y., M.L., S.W., and T.Z. performed the relevant cell experiments and contributed to the analysis and interpretation of data. Z.H., Q.Y., and J.C. drafted the article. Y.W., Y.Z., N.X., and T.C. critically revised important intellectual content. All authors critically reviewed the manuscript and approved the final version. All authors critically reviewed the manuscript and approved the final version.

DECLARATION OF INTERESTS

The authors declare no competing interests.

STAR★METHODS

Detailed methods are provided in the online version of this paper and include the following:

- KEY RESOURCES TABLE
- EXPERIMENTAL MODEL AND STUDY PARTICIPANT DETAILS
  - Cells and plasmids
  - Productions of SARS-CoV-2 PsV
  - Titration of SARS-CoV-2 PsV
  - Human plasma samples
  - Sera of spike protein-immunized hamsters
- METHOD DETAILS
  - SARS-CoV-2 PsV neutralization assays
  - Monoclonal antibodies and recombinant proteins
- QUANTIFICATION AND STATISTICAL ANALYSIS

SUPPLEMENTAL INFORMATION

Supplemental information can be found online at https://doi.org/10.1016/j.crmeth.2024.100856.

Received: February 5, 2024
Revised: June 18, 2024
Accepted: August 14, 2024
Published: September 6, 2024

REFERENCES

1. WHO (2024). WHO Coronavirus (COVID-19) Dashboard. https://data.who.int/dashboards/covid19/.
2. Uriu, K., Ito, J., Zahradnik, J., Fujita, S., Kosugi, Y., Schreiber, G., and Genotype to Phenotype Japan G2P-Japan Consortium; and Sato, K. (2023). Enhanced transmissibility, infectivity, and immune resistance of the SARS-CoV-2 omicron XBB.1.5 variant. Lancet Infect. Dis. *23*, 280–281. https://doi.org/10.1016/S1473-3099(23)00051-8.
3. Yamasoba, D., Uriu, K., Plianchaisuk, A., Kosugi, Y., Pan, L., Zahradnik, J., Genotype to Phenotype Japan G2P-Japan Consortium; Ito, J., and Sato, K. (2023). Virological characteristics of the SARS-CoV-2 omicron XBB.1.16 variant. Lancet Infect. Dis. *23*, 655–656. https://doi.org/10.1016/S1473-3099(23)00278-5.
4. Yue, C., Song, W., Wang, L., Jian, F., Chen, X., Gao, F., Shen, Z., Wang, Y., Wang, X., and Cao, Y. (2023). ACE2 binding and antibody evasion in enhanced transmissibility of XBB.1.5. Lancet Infect. Dis. *23*, 278–280. https://doi.org/10.1016/S1473-3099(23)00010-5.
5. Tamura, T., Ito, J., Uriu, K., Zahradnik, J., Kida, I., Anraku, Y., Nasser, H., Shofa, M., Oda, Y., Lytras, S., et al. (2023). Virological characteristics of the SARS-CoV-2 XBB variant derived from recombination of two Omicron subvariants. Nat. Commun. *14*, 2800. https://doi.org/10.1038/s41467-023-38435-3.
6. Cao, Y., Jian, F., Wang, J., Yu, Y., Song, W., Yisimayi, A., Wang, J., An, R., Chen, X., Zhang, N., et al. (2023). Imprinted SARS-CoV-2 humoral immunity induces convergent Omicron RBD evolution. Nature *614*, 521–529. https://doi.org/10.1038/s41586-022-05644-7.
7. Chang, L., Hou, W., Zhao, L., Zhang, Y., Wang, Y., Wu, L., Xu, T., Wang, L., Wang, J., Ma, J., et al. (2021). The prevalence of antibodies to SARS-CoV-2 among blood donors in China. Nat. Commun. *12*, 1383. https://doi.org/10.1038/s41467-021-21503-x.
8. Wibmer, C.K., Ayres, F., Hermanus, T., Madzivhandila, M., Kgagudi, P., Oosthuysen, B., Lambson, B.E., de Oliveira, T., Vermeulen, M., van der Berg, K., et al. (2021). SARS-CoV-2 501Y.V2 escapes neutralization by South African COVID-19 donor plasma. Nat. Med. *27*, 622–625. https://doi.org/10.1038/s41591-021-01285-x.
9. Xiong, H.L., Wu, Y.T., Cao, J.L., Yang, R., Liu, Y.X., Ma, J., Qiao, X.Y., Yao, X.Y., Zhang, B.H., Zhang, Y.L., et al. (2020). Robust neutralization

assay based on SARS-CoV-2 S-protein-bearing vesicular stomatitis virus (VSV) pseudovirus and ACE2-overexpressing BHK21 cells. Emerg. Microbes Infect. *9*, 2105–2113. https://doi.org/10.1080/22221751.2020.1815589.

10. Garcia-Beltran, W.F., St Denis, K.J., Hoelzemer, A., Lam, E.C., Nitido, A.D., Sheehan, M.L., Berrios, C., Ofoman, O., Chang, C.C., Hauser, B.M., et al. (2022). mRNA-based COVID-19 vaccine boosters induce neutralizing immunity against SARS-CoV-2 Omicron variant. Cell *185*, 457–466.e4. https://doi.org/10.1016/j.cell.2021.12.033.
11. Gilbert, P.B., Montefiori, D.C., McDermott, A.B., Fong, Y., Benkeser, D., Deng, W., Zhou, H., Houchens, C.R., Martins, K., Jayashankar, L., et al. (2022). Immune correlates analysis of the mRNA-1273 COVID-19 vaccine efficacy clinical trial. Science *375*, 43–50. https://doi.org/10.1126/science.abm3425.
12. Fong, Y., McDermott, A.B., Benkeser, D., Roels, S., Stieh, D.J., Vandebosch, A., Le Gars, M., Van Roey, G.A., Houchens, C.R., Martins, K., et al. (2022). Immune correlates analysis of the ENSEMBLE single Ad26.-COV2.S dose vaccine efficacy clinical trial. Nat. Microbiol. *7*, 1996–2010. https://doi.org/10.1038/s41564-022-01262-1.
13. Mykytyn, A.Z., Rissmann, M., Kok, A., Rosu, M.E., Schipper, D., Breugem, T.I., van den Doel, P.B., Chandler, F., Bestebroer, T., de Wit, M., et al. (2022). Antigenic cartography of SARS-CoV-2 reveals that Omicron BA.1 and BA.2 are antigenically distinct. Sci. Immunol. *7*, eabq4450. https://doi.org/10.1126/sciimmunol.abq4450.
14. Mykytyn, A.Z., Rosu, M.E., Kok, A., Rissmann, M., van Amerongen, G., Geurtsvankessel, C., de Vries, R.D., Munnink, B.B.O., Smith, D.J., Koopmans, M.P.G., et al. (2023). Antigenic mapping of emerging SARS-CoV-2 omicron variants BM.1.1.1, BQ.1.1, and XBB.1. Lancet. Microbe *4*, e294–e295. https://doi.org/10.1016/S2666-5247(22)00384-6.
15. Wilks, S.H., Mühlemann, B., Shen, X., Türeli, S., LeGresley, E.B., Netzl, A., Caniza, M.A., Chacaltana-Huarcaya, J.N., Corman, V.M., Daniell, X., et al. (2023). Mapping SARS-CoV-2 antigenic relationships and serological responses. Science *382*, eadj0070. https://doi.org/10.1126/science.adj0070.
16. Subach, O.M., Cranfill, P.J., Davidson, M.W., and Verkhusha, V.V. (2011). An enhanced monomeric blue fluorescent protein with the high chemical stability of the chromophore. PLoS One *6*, e28674. https://doi.org/10.1371/journal.pone.0028674.
17. Goedhart, J., von Stetten, D., Noirclerc-Savoye, M., Lelimousin, M., Joosen, L., Hink, M.A., van Weeren, L., Gadella, T.W.J., Jr., and Royant, A. (2012). Structure-guided evolution of cyan fluorescent proteins towards a quantum yield of 93. Nat. Commun. *3*, 751. https://doi.org/10.1038/ncomms1738.
18. Ai, H.W., Henderson, J.N., Remington, S.J., and Campbell, R.E. (2006). Directed evolution of a monomeric, bright and photostable version of Clavularia cyan fluorescent protein: structural characterization and applications in fluorescence imaging. Biochem. J. *400*, 531–540. https://doi.org/10.1042/BJ20060874.
19. Campbell, B.C., Nabel, E.M., Murdock, M.H., Lao-Peregrin, C., Tsoulfas, P., Blackmore, M.G., Lee, F.S., Liston, C., Morishita, H., and Petsko, G.A. (2020). mGreenLantern: a bright monomeric fluorescent protein with rapid expression and cell filling properties for neuronal imaging. Proc. Natl. Acad. Sci. USA *117*, 30710–30721. https://doi.org/10.1073/pnas.2000942117.
20. Hirano, M., Ando, R., Shimozono, S., Sugiyama, M., Takeda, N., Kurokawa, H., Deguchi, R., Endo, K., Haga, K., Takai-Todaka, R., et al. (2022). A highly photostable and bright green fluorescent protein. Nat. Biotechnol. *40*, 1132–1142. https://doi.org/10.1038/s41587-022-01278-2.
21. Shaner, N.C., Lambert, G.G., Chammas, A., Ni, Y., Cranfill, P.J., Baird, M.A., Sell, B.R., Allen, J.R., Day, R.N., Israelsson, M., et al. (2013). A bright monomeric green fluorescent protein derived from Branchiostoma lanceolatum. Nat. Methods *10*, 407–409. https://doi.org/10.1038/nmeth.2413.
22. Ai, H.W., Hazelwood, K.L., Davidson, M.W., and Campbell, R.E. (2008). Fluorescent protein FRET pairs for ratiometric imaging of dual biosensors. Nat. Methods *5*, 401–403. https://doi.org/10.1038/nmeth.1207.
23. Shcherbakova, D.M., Hink, M.A., Joosen, L., Gadella, T.W.J., and Verkhusha, V.V. (2012). An orange fluorescent protein with a large Stokes shift for single-excitation multicolor FCCS and FRET imaging. J. Am. Chem. Soc. *134*, 7913–7923. https://doi.org/10.1021/ja3018972.
24. Chu, J., Oh, Y., Sens, A., Ataie, N., Dana, H., Macklin, J.J., Laviv, T., Welf, E.S., Dean, K.M., Zhang, F., et al. (2016). A bright cyan-excitable orange fluorescent protein facilitates dual-emission microscopy and enhances bioluminescence imaging in vivo. Nat. Biotechnol. *34*, 760–767. https://doi.org/10.1038/nbt.3550.
25. Erdogan, M., Fabritius, A., Basquin, J., and Griesbeck, O. (2020). Targeted In Situ Protein Diversification and Intra-organelle Validation in Mammalian Cells. Cell Chem. Biol. *27*, 610–621.e5. https://doi.org/10.1016/j.chembiol.2020.02.004.
26. Bajar, B.T., Wang, E.S., Lam, A.J., Kim, B.B., Jacobs, C.L., Howe, E.S., Davidson, M.W., Lin, M.Z., and Chu, J. (2016). Improving brightness and photostability of green and red fluorescent proteins for live cell imaging and FRET reporting. Sci. Rep. *6*, 20889. https://doi.org/10.1038/srep20889.
27. Bindels, D.S., Haarbosch, L., van Weeren, L., Postma, M., Wiese, K.E., Mastop, M., Aumonier, S., Gotthard, G., Royant, A., Hink, M.A., and Gadella, T.W.J., Jr. (2017). mScarlet: a bright monomeric red fluorescent protein for cellular imaging. Nat. Methods *14*, 53–56. https://doi.org/10.1038/nmeth.4074.
28. Subach, O.M., Vlaskina, A.V., Agapova, Y.K., Dorovatovskii, P.V., Nikolaeva, A.Y., Ivashkina, O.I., Popov, V.O., Piatkevich, K.D., Khrenova, M.G., Smirnova, T.A., et al. (2021). LSSmScarlet, dCyRFP2s, dCyOFP2s and CRISPRed2s, Genetically Encoded Red Fluorescent Proteins with a Large Stokes Shift. Int. J. Mol. Sci. *22*, 12887. https://doi.org/10.3390/ijms222312887.
29. Yang, J., Wang, L., Yang, F., Luo, H., Xu, L., Lu, J., Zeng, S., and Zhang, Z. (2013). mBeRFP, an improved large stokes shift red fluorescent protein. PLoS One *8*, e64849. https://doi.org/10.1371/journal.pone.0064849.
30. Guan, Y., Meurer, M., Raghavan, S., Rebane, A., Lindquist, J.R., Santos, S., Kats, I., Davidson, M.W., Mazitschek, R., Hughes, T.E., et al. (2015). Live-cell multiphoton fluorescence correlation spectroscopy with an improved large Stokes shift fluorescent protein. Mol. Biol. Cell *26*, 2054–2066. https://doi.org/10.1091/mbc.E14-10-1473.
31. Matlashov, M.E., Shcherbakova, D.M., Alvelid, J., Baloban, M., Pennacchietti, F., Shemetov, A.A., Testa, I., and Verkhusha, V.V. (2020). A set of monomeric near-infrared fluorescent proteins for multicolor imaging across scales. Nat. Commun. *11*, 239. https://doi.org/10.1038/s41467-019-13897-6.
32. Shcherbakova, D.M., and Verkhusha, V.V. (2013). Near-infrared fluorescent proteins for multicolor in vivo imaging. Nat. Methods *10*, 751–754. https://doi.org/10.1038/nmeth.2521.
33. Oliinyk, O.S., Shemetov, A.A., Pletnev, S., Shcherbakova, D.M., and Verkhusha, V.V. (2019). Smallest near-infrared fluorescent protein evolved from cyanobacteriochrome as versatile tag for spectral multiplexing. Nat. Commun. *10*, 279. https://doi.org/10.1038/s41467-018-08050-8.
34. Wang, S., Sun, H., Zhang, Y., Yuan, L., Wang, Y., Zhang, T., Wang, S., Zhang, J., Yu, H., Xiong, H., et al. (2022). Three SARS-CoV-2 antibodies provide broad and synergistic neutralization against variants of concern, including Omicron. Cell Rep. *39*, 110862. https://doi.org/10.1016/j.celrep.2022.110862.
35. Tortorici, M.A., Beltramello, M., Lempp, F.A., Pinto, D., Dang, H.V., Rosen, L.E., McCallum, M., Bowen, J., Minola, A., Jaconi, S., et al. (2020). Ultrapotent human antibodies protect against SARS-CoV-2 challenge via multiple mechanisms. Science *370*, 950–957. https://doi.org/10.1126/science.abe3354.

36. Brouwer, P.J.M., Caniels, T.G., van der Straten, K., Snitselaar, J.L., Aldon, Y., Bangaru, S., Torres, J.L., Okba, N.M.A., Claireaux, M., Kerster, G., et al. (2020). Potent neutralizing antibodies from COVID-19 patients define multiple targets of vulnerability. Science *369*, 643–650. https://doi.org/10.1126/science.abc5902.

37. Chi, X., Yan, R., Zhang, J., Zhang, G., Zhang, Y., Hao, M., Zhang, Z., Fan, P., Dong, Y., Yang, Y., et al. (2020). A neutralizing human antibody binds to the N-terminal domain of the Spike protein of SARS-CoV-2. Science *369*, 650–655. https://doi.org/10.1126/science.abc6952.

38. Baum, A., Fulton, B.O., Wloga, E., Copin, R., Pascal, K.E., Russo, V., Giordano, S., Lanza, K., Negron, N., Ni, M., et al. (2020). Antibody cocktail to SARS-CoV-2 spike protein prevents rapid mutational escape seen with individual antibodies. Science *369*, 1014–1018. https://doi.org/10.1126/science.abd0831.

39. Westendorf, K., Žentelis, S., Wang, L., Foster, D., Vaillancourt, P., Wiggin, M., Lovett, E., van der Lee, R., Hendle, J., Pustilnik, A., et al. (2022). LY-CoV1404 (bebtelovimab) potently neutralizes SARS-CoV-2 variants. Cell Rep. *39*, 110812. https://doi.org/10.1016/j.celrep.2022.110812.

40. Wu, Y., Wang, S., Zhang, Y., Yuan, L., Zheng, Q., Wei, M., Shi, Y., Wang, Z., Ma, J., Wang, K., et al. (2022). Lineage-mosaic and mutation-patched spike proteins for broad-spectrum COVID-19 vaccine. Cell Host Microbe *30*, 1732–1744.e7. https://doi.org/10.1016/j.chom.2022.10.011.

41. Loo, Y.M., McTamney, P.M., Arends, R.H., Abram, M.E., Aksyuk, A.A., Diallo, S., Flores, D.J., Kelly, E.J., Ren, K., Roque, R., et al. (2022). The SARS-CoV-2 monoclonal antibody combination, AZD7442, is protective in nonhuman primates and has an extended half-life in humans. Sci. Transl. Med. *14*, eabl8124. https://doi.org/10.1126/scitranslmed.abl8124.

42. Planas, D., Staropoli, I., Michel, V., Lemoine, F., Donati, F., Prot, M., Porrot, F., Guivel-Benhassine, F., Jeyarajah, B., Brisebarre, A., et al. (2024). Distinct evolution of SARS-CoV-2 Omicron XBB and BA.2.86 lineages combining increased fitness and antibody evasion. Preprint at bioRxiv. https://doi.org/10.1101/2023.11.20.567873.

43. Hu, Y., Zou, J., Kurhade, C., Deng, X., Chang, H.C., Kim, D.K., Shi, P.Y., Ren, P., and Xie, X. (2023). Less neutralization evasion of SARS-CoV-2 BA.2.86 than XBB sublineages and CH.1.1. Emerg. Microbes Infect. *12*, 2271089. https://doi.org/10.1080/22221751.2023.2271089.

44. Khan, K., Lustig, G., Römer, C., Reedoy, K., Jule, Z., Karim, F., Ganga, Y., Bernstein, M., Baig, Z., Jackson, L., et al. (2023). Evolution and neutralization escape of the SARS-CoV-2 BA.2.86 subvariant. Nat. Commun. *14*, 8078. https://doi.org/10.1038/s41467-023-43703-3.

45. Wang, Q., Guo, Y., Liu, L., Schwanz, L.T., Li, Z., Nair, M.S., Ho, J., Zhang, R.M., Iketani, S., Yu, J., et al. (2023). Antigenicity and receptor affinity of SARS-CoV-2 BA.2.86 spike. Nature *624*, 639–644. https://doi.org/10.1038/s41586-023-06750-w.

46. Tan, C.W., Chia, W.N., Qin, X., Liu, P., Chen, M.I.C., Tiu, C., Hu, Z., Chen, V.C.W., Young, B.E., Sia, W.R., et al. (2020). A SARS-CoV-2 surrogate virus neutralization test based on antibody-mediated blockage of ACE2-spike protein-protein interaction. Nat. Biotechnol. *38*, 1073–1078. https://doi.org/10.1038/s41587-020-0631-z.

47. Liu, H., Varvel, S., Chen, G., McConnell, J., Caffrey, R., Galdzicka, M., and Shabahang, S. (2022). Simultaneous measurement of multiple variant-specific SARS-CoV-2 neutralizing antibodies with a multiplexed flow cytometric assay. Front. Immunol. *13*, 1039163. https://doi.org/10.3389/fimmu.2022.1039163.

48. Fenwick, C., Turelli, P., Pellaton, C., Farina, A., Campos, J., Raclot, C., Pojer, F., Cagno, V., Nusslé, S.G., D'Acremont, V., et al. (2021). A high-throughput cell- and virus-free assay shows reduced neutralization of SARS-CoV-2 variants by COVID-19 convalescent plasma. Sci. Transl. Med. *13*, eabi8452. https://doi.org/10.1126/scitranslmed.abi8452.

49. Pinto, D., Park, Y.J., Beltramello, M., Walls, A.C., Tortorici, M.A., Bianchi, S., Jaconi, S., Culap, K., Zatta, F., De Marco, A., et al. (2020). Cross-neutralization of SARS-CoV-2 by a human monoclonal SARS-CoV antibody. Nature *583*, 290–295. https://doi.org/10.1038/s41586-020-2349-y.

50. Yuan, M., Wu, N.C., Zhu, X., Lee, C.C.D., So, R.T.Y., Lv, H., Mok, C.K.P., and Wilson, I.A. (2020). A highly conserved cryptic epitope in the receptor binding domains of SARS-CoV-2 and SARS-CoV. Science *368*, 630–633. https://doi.org/10.1126/science.abb7269.

51. Duan, X., Shi, R., Liu, P., Huang, Q., Wang, F., Chen, X., Feng, H., Huang, W., Xiao, J., and Yan, J. (2022). A non-ACE2-blocking neutralizing antibody against Omicron-included SARS-CoV-2 variants. Signal Transduct. Target. Ther. *7*, 23. https://doi.org/10.1038/s41392-022-00879-2.

52. Pinto, D., Sauer, M.M., Czudnochowski, N., Low, J.S., Tortorici, M.A., Housley, M.P., Noack, J., Walls, A.C., Bowen, J.E., Guarino, B., et al. (2021). Broad betacoronavirus neutralization by a stem helix-specific human antibody. Science *373*, 1109–1116. https://doi.org/10.1126/science.abj3321.

53. Zhang, Y., Wang, S., Wu, Y., Hou, W., Yuan, L., Shen, C., Wang, J., Ye, J., Zheng, Q., Ma, J., et al. (2021). Virus-Free and Live-Cell Visualizing SARS-CoV-2 Cell Entry for Studies of Neutralizing Antibodies and Compound Inhibitors. Small Methods *5*, 2001031. https://doi.org/10.1002/smtd.202001031.

54. Sievers, B.L., Gelbart, T., and Tan, G.S. (2022). A high-throughput SARS-CoV-2 pseudovirus multiplex neutralization assay. STAR Protoc. *3*, 101835. https://doi.org/10.1016/j.xpro.2022.101835.

55. Sievers, B.L., Chakraborty, S., Xue, Y., Gelbart, T., Gonzalez, J.C., Cassidy, A.G., Golan, Y., Prahl, M., Gaw, S.L., Arunachalam, P.S., et al. (2022). Antibodies elicited by SARS-CoV-2 infection or mRNA vaccines have reduced neutralizing activity against Beta and Omicron pseudoviruses. Sci. Transl. Med. *14*, eabn7842. https://doi.org/10.1126/scitranslmed.abn7842.

56. Lingemann, M., Amaro-Carambot, E., Lamirande, E.W., Pierson, T.C., and Whitehead, S.S. (2024). Simultaneous quantitation of neutralizing antibodies against all four dengue virus serotypes using optimized reporter virus particles. J. Virol. *98*, e0068124. https://doi.org/10.1128/jvi.00681-24.

57. Wang, Q., Iketani, S., Li, Z., Liu, L., Guo, Y., Huang, Y., Bowen, A.D., Liu, M., Wang, M., Yu, J., et al. (2023). Alarming antibody evasion properties of rising SARS-CoV-2 BQ and XBB subvariants. Cell *186*, 279–286.e88. https://doi.org/10.1016/j.cell.2022.12.018.

58. Chen, S., Huang, Z., Guo, Y., Guo, H., Jian, L., Xiao, J., Yao, X., Yu, H., Cheng, T., Zhang, Y., et al. (2023). Evolving spike mutations in SARS-CoV-2 Omicron variants facilitate evasion from breakthrough infection-acquired antibodies. Cell Discov. *9*, 86. https://doi.org/10.1038/s41421-023-00584-6.

59. Wang, X., Jiang, S., Jiang, S., Li, X., Ai, J., Lin, K., Lv, S., Zhang, S., Li, M., Li, J., et al. (2023). Neutralization of SARS-CoV-2 BQ.1.1, CH.1.1, and XBB.1.5 by breakthrough infection sera from previous and recent waves in China. Cell Discov. *9*, 64. https://doi.org/10.1038/s41421-023-00569-5.

60. Kaku, Y., Okumura, K., Padilla-Blanco, M., Kosugi, Y., Uriu, K., Hinay, A.A., Jr., Chen, L., Plianchaisuk, A., Kobiyama, K., Ishii, K.J., et al. (2024). Virological characteristics of the SARS-CoV-2 JN.1 variant. Lancet Infect. Dis. *24*, e82. https://doi.org/10.1016/S1473-3099(23)00813-7.

61. Mathieu, E., Ritchie, H., Ortiz-Ospina, E., Roser, M., Hasell, J., Appel, C., Giattino, C., and Rodés-Guirao, L. (2021). A global database of COVID-19 vaccinations. Nat. Hum. Behav. *5*, 947–953. https://doi.org/10.1038/s41562-021-01122-8.

62. Muhlemann, B., Trimpert, J., Walper, F., Schmidt, M.L., Jansen, J., Schroeder, S., Jeworowski, L.M., Beheim-Schwarzbach, J., Bleicker, T., Niemeyer, D., et al. (2024). Antigenic cartography using variant-specific hamster sera reveals substantial antigenic variation among Omicron subvariants. Proc. Natl. Acad. Sci. USA *121*, e2310917121. https://doi.org/10.1073/pnas.2310917121.

63. Zhang, Y., Wei, M., Wu, Y., Wang, J., Hong, Y., Huang, Y., Yuan, L., Ma, J., Wang, K., Wang, S., et al. (2022). Cross-species tropism and antigenic

landscapes of circulating SARS-CoV-2 variants. Cell Rep. *38*, 110558. https://doi.org/10.1016/j.celrep.2022.110558.

64. Wu, Y., Huang, X., Yuan, L., Wang, S., Zhang, Y., Xiong, H., Chen, R., Ma, J., Qi, R., Nie, M., et al. (2021). A recombinant spike protein subunit vaccine confers protective immunity against SARS-CoV-2 infection and transmission in hamsters. Sci. Transl. Med. *13*, eabg1143. https://doi.org/10.1126/scitranslmed.abg1143.

65. Hsieh, C.L., Goldsmith, J.A., Schaub, J.M., DiVenere, A.M., Kuo, H.C., Javanmardi, K., Le, K.C., Wrapp, D., Lee, A.G., Liu, Y., et al. (2020). Structure-based design of prefusion-stabilized SARS-CoV-2 spikes. Science *369*, 1501–1505. https://doi.org/10.1126/science.abd0826.

STAR★METHODS

KEY RESOURCES TABLE

REAGENT or RESOURCE	SOURCE	IDENTIFIER
**Antibodies**		
S2M11	Tortorici et al., 2020[35]	N/A
COVA2-15	Brouwer et al., 2020[36]	N/A
LY-CoV1404	Westendorf et al., 2022[39]	N/A
XMA01	Wang et al., 2022[34]	N/A
4A8	Chi et al., 2020[37]	N/A
REGN10933	Baum et al., 2020[38]	N/A
36H6	Wu et al., 2022[40]	N/A
COV2-2130	Loo et al., 2022[41]	N/A
BD55-5514	Cao et al., 2023[6]	N/A
85F7	Wu et al., 2022[40]	N/A
BD55-5840	Cao et al., 2023[6]	N/A
**Bacterial and virus strains**		
D614G pseudovirus	Zhang et al., 2022[63]	N/A
B.1.351 (Beta) pseudovirus	Zhang et al., 2022[63]	N/A
B.1.617.2 (Delta) pseudovirus	Zhang et al., 2022[63]	N/A
BA.1 pseudovirus	Wu et al., 2022[40]	N/A
BA.2 pseudovirus	Wu et al., 2022[40]	N/A
BA.5 pseudovirus	Wu et al., 2022[40]	N/A
BQ.1.1 pseudovirus	Chen et al., 2023[58]	N/A
XBB pseudovirus	Chen et al., 2023[58]	N/A
CH.1.1 pseudovirus	Chen et al., 2023[58]	N/A
DY.1.1 pseudovirus	This paper	N/A
BF.7 pseudovirus	This paper	N/A
BQ.1.1.35 pseudovirus	This paper	N/A
BQ.1.1.71 pseudovirus	This paper	N/A
ER.1.1 pseudovirus	This paper	N/A
EY.1 pseudovirus	This paper	N/A
FA.1 pseudovirus	This paper	N/A
DV.1.1 pseudovirus	This paper	N/A
FK.1 pseudovirus	This paper	N/A
CH.1.1.6 pseudovirus	This paper	N/A
CH.1.1.16 pseudovirus	This paper	N/A
CH.1.1.2 pseudovirus	This paper	N/A
XBB.2.3.2 pseudovirus	This paper	N/A
XBB.1.28 pseudovirus	This paper	N/A
XBB.1.5.27 pseudovirus	This paper	N/A
XBB.1.16 pseudovirus	This paper	N/A
XBB.1.12 pseudovirus	This paper	N/A
FZ.1 pseudovirus	This paper	N/A
XBB.1.5 pseudovirus	This paper	N/A
XBB.1.5.30 pseudovirus	This paper	N/A
XBB.1.5.3 pseudovirus	This paper	N/A
XBB.1.5.12 pseudovirus	This paper	N/A

(*Continued on next page*)

Continued

REAGENT or RESOURCE	SOURCE	IDENTIFIER
XBB.1.5.1 pseudovirus	This paper	N/A
FD.3 pseudovirus	This paper	N/A
XBB.1.17.1 pseudovirus	This paper	N/A
XBB.1.5.2 pseudovirus	This paper	N/A
EG.5 pseudovirus	This paper	N/A
EG.1 pseudovirus	This paper	N/A
XBB.1.5.4 pseudovirus	This paper	N/A
EK.1 pseudovirus	This paper	N/A
FG.1 pseudovirus	This paper	N/A
XBB.1.8 pseudovirus	This paper	N/A
XBB.1.36 pseudovirus	This paper	N/A
XBB.1.31 pseudovirus	This paper	N/A
HV.1 pseudovirus	This paper	N/A
HK.3 pseudovirus	This paper	N/A
FL.15.1.1 pseudovirus	This paper	N/A
DS.1 pseudovirus	This paper	N/A
BA.2.75 pseudovirus	This paper	N/A
XBF.7.1 pseudovirus	This paper	N/A
XAY.1.1.1 pseudovirus	This paper	N/A
JN.1 pseudovirus	This paper	N/A
BA.2.86 pseudovirus	This paper	N/A
CM.8.1.1 pseudovirus	This paper	N/A
Biological samples		
COVID-19 vaccinated participants plasma samples	Chen et al., 2023[58]	N/A
COVID-19 human convalescent plasma samples	Chen et al., 2023[58]	N/A
Chemicals, peptides, and recombinant proteins		
Recombinant human ACE2 (human Fc tag)	Chen et al., 2023[58]	N/A
Experimental models: Cell lines		
ExpiCHO-S cells	Thermo Scientific	Cat# A29127
293T/17 cells	ATCC	Cat# CRL-11268
H1299-hACE2 (human ACE2)	This paper	N/A
Recombinant DNA		
Plasmid: EIRBsMie-hACE2	Wu et al., 2022[40]	N/A
Plasmid: pLVmie-iRFP670	This paper	N/A
Plasmid: pLVmie-mRuby3	This paper	N/A
Plasmid: pLVmie-mNeonGreen	This paper	N/A
Plasmid: pLVmie-mTagBFP2	This paper	N/A
Plasmid: pLVEF1α-iRFP670	This paper	N/A
Plasmid: pLVEF1α-mRuby3	This paper	N/A
Plasmid: pLVEF1α-mNeonGreen	This paper	N/A
Plasmid: pLVEF1α-mTagBFP2	This paper	N/A
Plasmid: pLVEF1α-emiRFP713	This paper	N/A
Plasmid: pLVEF1α-emiRFP703	This paper	N/A
Plasmid: pLVEF1α-iRFP670	This paper	N/A
Plasmid: pLVEF1α-miRFP670nano	This paper	N/A
Plasmid: pLVEF1α-hmKeima8.5	This paper	N/A
Plasmid: pLVEF1α-mBeRFP	This paper	N/A
Plasmid: pLVEF1α-LSSmScarlet	This paper	N/A

(*Continued on next page*)

Continued		
REAGENT or RESOURCE	SOURCE	IDENTIFIER
Plasmid: pLVEF1α-mScarlet-I	This paper	N/A
Plasmid: pLVEF1α-mCRISPRed	This paper	N/A
Plasmid: pLVEF1α-CyOFP1	This paper	N/A
Plasmid: pLVEF1α-LSSmOrange	This paper	N/A
Plasmid: pLVEF1α-mAmetrine	This paper	N/A
Plasmid: pLVEF1α-mNeonGreen	This paper	N/A
Plasmid: pLVEF1α-StayCold	This paper	N/A
Plasmid: pLVEF1α-mTFP1	This paper	N/A
Plasmid: pLVEF1α-mTurquoise2	This paper	N/A
Software and algorithms		
Columbus Analysis system (version 2.5.0)	PerkinElmer	https://www.perkinelmer.com/
GraphPad Prism (version 9.5.1)	Graphpad	https://www.graphpad.com/
R software (version 4.3.2)	R Foundation	https://www.r-project.org/
Origin (version 9.9.0.225)	OriginLab	https://www.originlab.com/

EXPERIMENTAL MODEL AND STUDY PARTICIPANT DETAILS

Cells and plasmids

Recombinant spike proteins were produced in ExpiCHO-S Cells using the ExpiCHO Expression System (Thermo Fisher). The HEK 293T/17 (ATCC Cat# CRL-11268) cells were used for lentiviral-based PsV production. The H1299-huACE2 cell, which stably expresses human ACE2 used for SARS-CoV-2 PsV infection, was developed using lentiviral transduction as previously described.[53] The lentiviral vector for generating the H1299-huACE2 stable cells was constructed via ligation of ACE2 cDNA into the pLV-EF1α-MCS-IRES-Bsd vector. The H1299-hACE2 cells were cultured using Dulbecco's Modified Eagle Medium containing 10% fetal bovine serum (FBS) supplemented with blasticidin (10 μg/mL). For lentiviral PsV reporter vectors, the initial pLVEF1α-mNG plasmid was constructed in our previous study.[7] Human codon-optimized expression sequences of mTagBFP2, mTurquoise2, mTFP1, mGreenLantern, Staygold, mAmetrine, LSSmOrange, CyOFP1, mCRISPRred, mRuby3, mScarlet-I, LSSmScarlet-I, mBeRFP, hmKemia8.5, emiRFP670, miRFP670nano, emiRFP703 and emiRFP713 were synthesized (Generalbiol, Anhui, China) and ligated into the pLVEF1α-mNG with replacements of the mNeonGreen to generate various pLVEF1α-FP vectors. The hCMVmie promoter from the EIRBsMie vector was cloned into the pLVEF1α-mNG, pLVEF1α-mTagBFP2, pLVEF1α-mRuby3, and pLVEF1α-iRFP670 with replacement of the EF1α promoter to construct the corresponding pLVMie-FP vectors, respectively. For plasmids expressing different spike variants, the corresponding spike-expressing cassettes (the C-terminal 18 aa was replaced as a HiBit Tag) were generated via site-directed site-specific mutagenesis on the EIRBsMie vector containing codon-optimized spike gene.[63] Expression plasmids of recombinant spike ectodomain proteins (aa 1 to 1207, referring to the MN908947.3) from BA.5 (EPI_ISL_11017528), BQ.1.1 (EPI_ISL_15514723), CH.1.1 (EPI_ISL_15345176), and XBB.1.5 (EPI_ISL_16818665) were constructed following previously described.[64] Notably, for all recombinant spike proteins, the furin-like cleavage site was removed (RRAR mutated to GSAS), and HexaPro stabilization mutations and C-terminal polyhistidine were introduced.[65]

Productions of SARS-CoV-2 PsV

We produce lentiviral-based SARS-CoV-2 PsV bearing different spike variants as previously described.[63] The detailed PsV production protocols using the Hieff-Trans·universal transfection reagent (Yeasen, China) or Lipofectamine3000 (Thermo Fisher) are described as follows.

(1) Seed HEK 293T/17 cells in 6-well plates at a density of $1x10^6$ cells per well-using growth medium comprising 10% fetal bovine serum (FBS) and 1× Dulbecco's Modified Eagle Medium (DMEM).
(2) Incubate the cells for approximately 18 h at 37°C with 5% CO_2.
(3) Prepare Mixture A by combining 750 ng of spike-expressing plasmid, 750 ng of psPAX2, 1.5 μg of shuttle vector carrying the fluorescence protein reporter (FP-reporter), 5 μL of Universal-A (or P3000) reagent, and 250 μL of Opti-MEM. Prepare Mixture B by combining 5 μL of Universal-B (or Lipofectamine 3000) reagent and 250 μL of Opti-MEM.
(4) Combine Mixture A with Mixture B, invert to mix, and incubate at room temperature for 5 min. Then, add 500 μL of the combined mixture to each well.
(5) After 4–6 h of incubation with the mixture, replace the media with a fresh growth medium. Continue incubating the cells at 37°C with 5% CO_2.

(6) After transfecting for 48 h, collect the supernatant containing infectious pseudovirus (PsV) from the 6-well plates into a 50 mL tube (temporarily stored at 4°C). Then, refresh the culture medium for the cells
(7) Collect the supernatant again from the 6-well plates after 72 h post-transfection into the same 50 mL tube. Filter the collected supernatant using a 0.45 μm pore-size filter.
(8) Aliquot the virus and store at −80°C until use.

Titration of SARS-CoV-2 PsV

This section describes the titration of PsV on H1299-ACE2 cells. The data generated from this procedure will enable users to accurately determine the virus titer (FFU/mL) used in neutralization experiments. The specific steps are as follows.

(1) Seed the 96-well plates with H1299-huACE2 at 6,000 cells per well in cell growth media (10% FBS, 1 x DMEM).
(2) Grow cells in the incubator overnight with 37°C and 5% CO_2.
(3) Dilute the virus in a medium containing 2% inactivated FBS and 1× DMEM in a 96-well plate. Begin with 20 μL of the virus in the first well and perform serial two-fold dilutions across eight gradients, then incubate at 37°C for 1 hour. The total volume in each well should be 120 μL.
(4) Add 100 μL PsV mixtures to H1299-hACE2 cells pre-seeded in 96-well plates. The cell plates were further cultured at 37°C in a CO_2 incubator.
(5) After 48h, image the plates using high-content imaging systems such as the Opera Phenix or Operetta CLS (PerkinElmer) for blue, green, red, and near-infrared fluorescence channels. Determine the number of PsV-infected cells (activated by mTagBFP2, mNeonGreen, mRuby3, or iRFP670) per well using the Columbus Image Analysis System (PerkinElmer).
(6) Generate a fitting curve (linear regression model) based on the amount of virus used in each well and the number of fluorescence points counted to determine the infectious PsV titers (FFU/well).

Human plasma samples

Plasma samples from humans who received COVID-19 vaccinations or who had recovered from SARS-CoV-2 infections involved in this study were described in our previous studies.[58] Written informed consent was obtained for each participant. This study was approved by the institutional review board of Huashan Hospital and School of Public Health (Xiamen University) following the Declaration of Helsinki.

Sera of spike protein-immunized hamsters

Lakeview Golden (LVG) Syrian hamsters were purchased from Charles River Laboratories (Beijing). The animals were fed in Specific-pathogen-free circumstances. The hamster studies were carried out in strict accordance with the recommendations of the Guide for the Care and Use of Laboratory Animals under the approval of the Institutional Animal Care and Use Committee of Xiamen University. Briefly, 6-to-8-week-old hamsters were used to evaluate the immunogenicity of the spike protein variants. For each protein, eight animals (half males and half females) were immunized intramuscularly twice with the spike proteins at 5μg per dose adjuvanted with FH002C in 200 μL, following a schedule of one priming plus one booster at weeks 3. Immunized hamsters' sera were collected at week 2 after the booster administration to measure the antibody titers.

METHOD DETAILS

SARS-CoV-2 PsV neutralization assays

The classical mFluo SARS-CoV-2 PsV neutralization assay was performed following previous studies.[7,58,63] The brief protocols for the quantitative fluorescence (qFluo) assay are as follows.

(1) Seed the 96-well plates with H1299-huACE2 at 6000 cells per well in cell growth media (10% FBS, 1 x DMEM).
(2) Grow cells in the incubator overnight with 37°C and 5% CO_2.
(3) According to the PsV titers, mix four spike-variant pseudoviruses (PsV), each carrying a reporter: mTagBFP, mNeonGreen, mRuby3, or iRFP670. Each virus should infect 1,000 to 1,500 cells per well. The total volume per well is 60 μL, with the remaining volume supplemented by medium (2% inactivated FBS, 1× DMEM).
(4) Gradient dilute samples (mAbs or plasmas) with medium (2% inactivated FBS, 1× DMEM), ensuring a total volume of 60 μL per well.
(5) Mix 60 μL of the PsV mixture with 60 μL of the sample dilution and incubate at 37°C for 1 h.
(6) Add 100 μL mixtures to H1299-hACE2 cells pre-seeded in 96-well plates. The cell plates were further cultured at 37°C in a CO_2 incubator.
(7) After 48h, the plates were imaged using the high-content imaging systems of Opera Phenix or Operetta CLS (PerkinElmer) for the blue, green, red, and near-infrared fluorescence channels. Determine the number of PsV-infected cells per well using the Columbus Image Analysis System (PerkinElmer).

(8) Subsequently, the infection inhibition ratio of each sample at different dilutions was calculated by comparing it with the PsV-only control wells. The IC_{50} (neutralization potency for a mAb or protein) or ID_{50} (for plasmas or sera) was defined as the concentration or dilution at which the relative infection cell numbers were reduced by 50% compared with the mean values of control wells. The IC_{50} or ID_{50} was determined by the 4PL regression using GraphPad Prism (version 9.5.1).

Monoclonal antibodies and recombinant proteins

Monoclonal antibodies tested in this study were constructed and produced in our laboratory according to their sequences described elsewhere. For each antibody, the codon-optimized variable genes were synthesized (Generalbiol, Anhui, China) and cloned into the plasmid (EIRBdMie), a dual-promoter vector containing constant regions of human IgG1 heavy and light chains. Antibodies were expressed in ExpiCHO-S cells via transfection of the EIRBdMie-based expressing plasmids using ExpiFectamine CHO Transfection Kit (Thermo Fisher). Transfected cells were cultured at 37°C with shaking at 125 RPM and 8% CO_2 in a stackable CO_2 incubator shaker. On day 5, supernatants were collected and purified using MabSelect PrismA resins (Cytiva). Recombinant spike ectodomain proteins were also produced in ExpiCHO-S cells and purified using Ni-NTA affinity chromatography as previously described.[64] The rhuACE2 protein was produced and purified following our previous study.[63]

QUANTIFICATION AND STATISTICAL ANALYSIS

The UMAP algorithm was employed to cluster the data of fluorescence expression levels of cells infected with pseudotyped virus. Prior to clustering, a normalization procedure was applied to the data. The Friedman test with Dunn's correction was applied to analyze differences among groups. The Spearman rank correlation coefficient was used for linear correlation analysis between the antibody titers. Statistical differences were considered to be significant for two-tailed p values of <0.05. UMAP clustering was conducted by R software (version 4.3.2). Statistical analyses were conducted by GraphPad Prism (version 9.5.1) and Origin (version 9.9.0.225). Relative neutralizing antibody titers (rNTs) are defined as the multiples of titers of immune antigen, such as in the serum of BA.5 immunized hamsters, where the rNT of BF.7 is calculated by dividing the neutralizing antibody titer of BF.7 by that of BA.5. Antigenic cartography maps were constructed with the R package "Racmacs" in R (version 4.3.2) based on the matrix of neutralization titer of serum. The number of optimizations was set to 1000.

Cell Reports Methods, Volume *4*

Supplemental information

A quadri-fluorescence SARS-CoV-2 pseudovirus system for efficient antigenic characterization of multiple circulating variants

Jijing Chen (陈积璟), Zehong Huang (黄泽宏), Jin Xiao (肖瑾), Shuangling Du (杜双伶), Qingfang Bu (布庆芳), Huilin Guo (郭慧琳), Jianghui Ye (叶江辉), Shiqi Chen (陈诗琦), Jiahua Gao (高佳华), Zonglin Li (李宗霖), Miaolin Lan (蓝妙琳), Shaojuan Wang (王邵娟), Tianying Zhang (张天英), Jiming Zhang (张继明), Yangtao Wu (巫洋涛), Yali Zhang (张雅丽), Ningshao Xia (夏宁邵), Quan Yuan (袁权), and Tong Cheng (程通)

Supplementary Figures

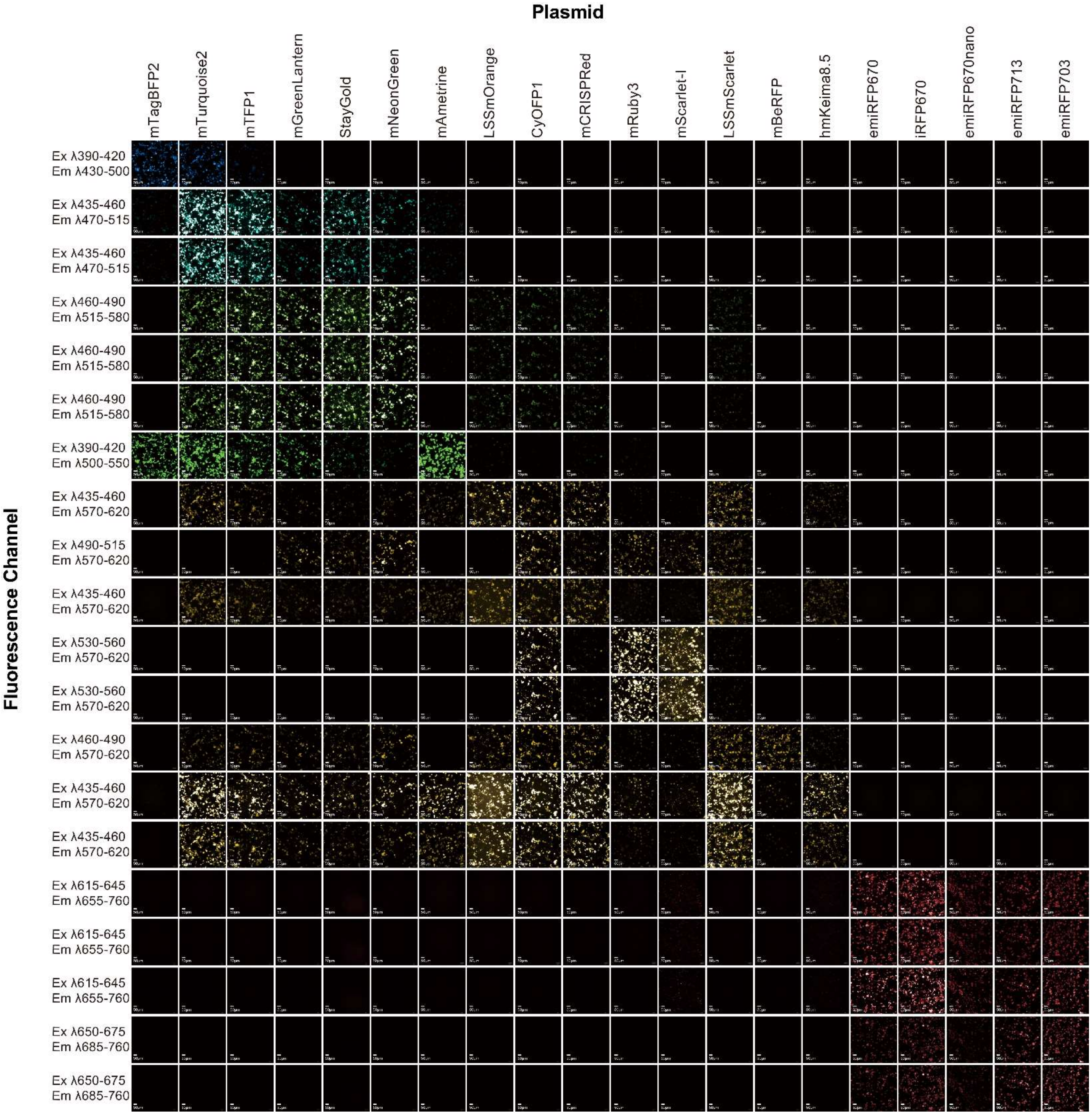

Figure S1 | The fluorescence imaging of 20 fluorescent proteins used for screening in different fluorescence channels, related to the middle panel of Figure 1A.

A field-of-view of fluorescence imaging of 293T/17 cells transfected with each fluorescent plasmid for 48h. The left side is marked with the excitation wavelength (Ex) and emission wavelength (Em) corresponding to each fluorescence channel.

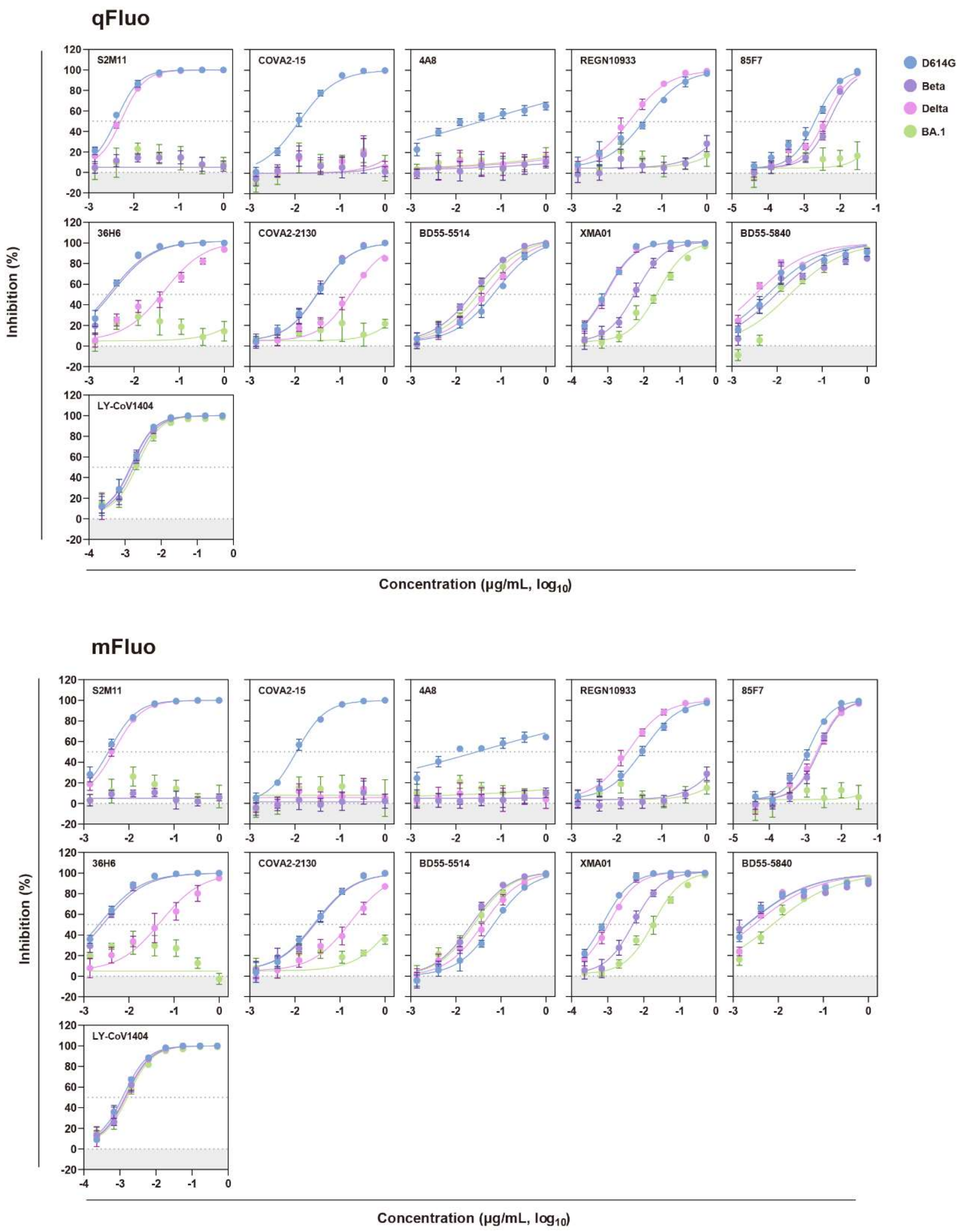

Figure S2 | Neutralization profiles for 11 mAbs detected by qFluo and mFluo systems, related to the left panel of Figure 4A.

All mAbs were tested at 3-fold serial dilutions. The data were plotted as the mean value and SD of ≥3 technical replicates.

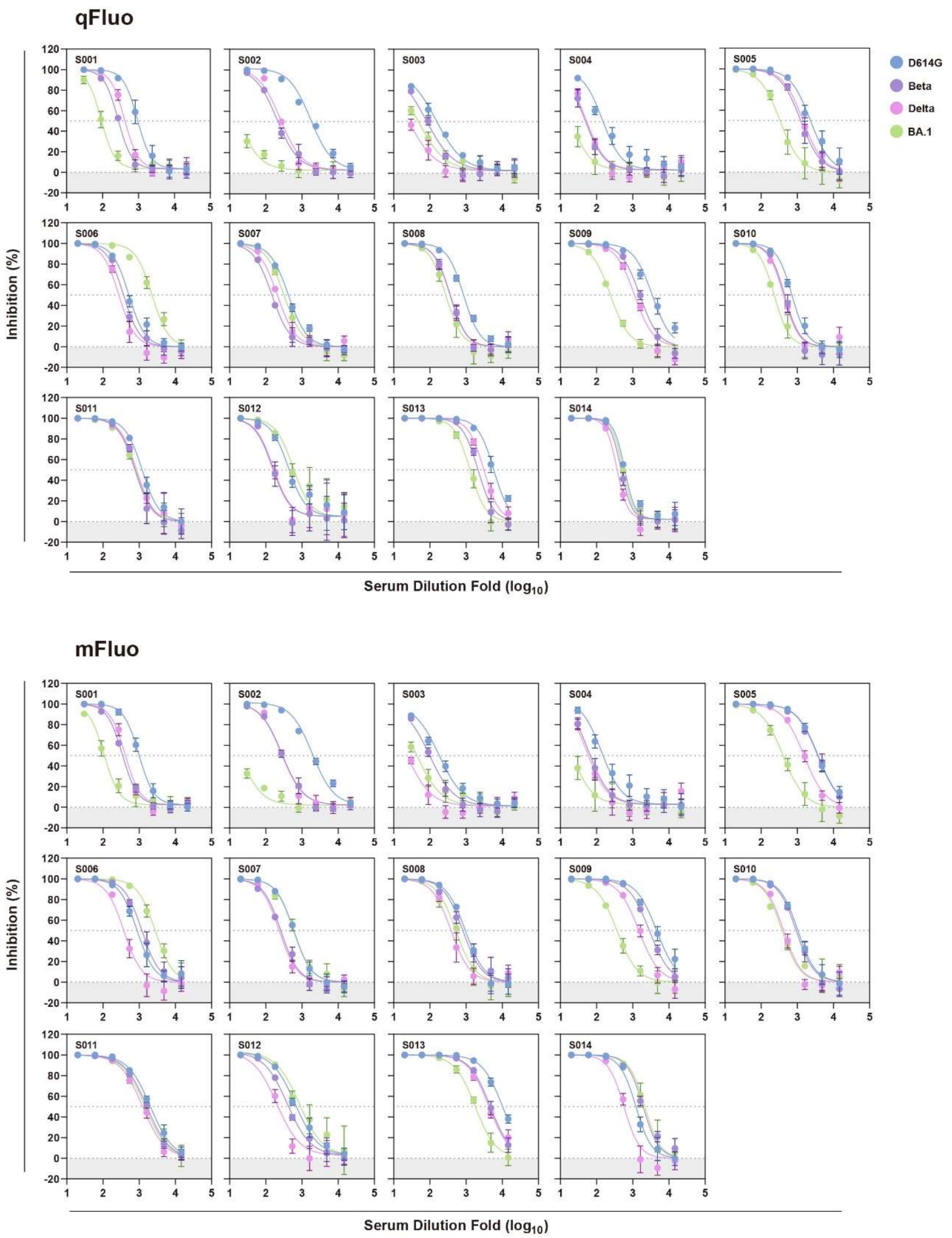

Figure S3 | Neutralization profiles for 14 plasmas detected by qFluo and mFluo systems, related to the right panel of Figure 4A.

All sera were tested at 3-fold serial dilutions. The data were plotted as the mean value and SD of ≥3 technical replicates.

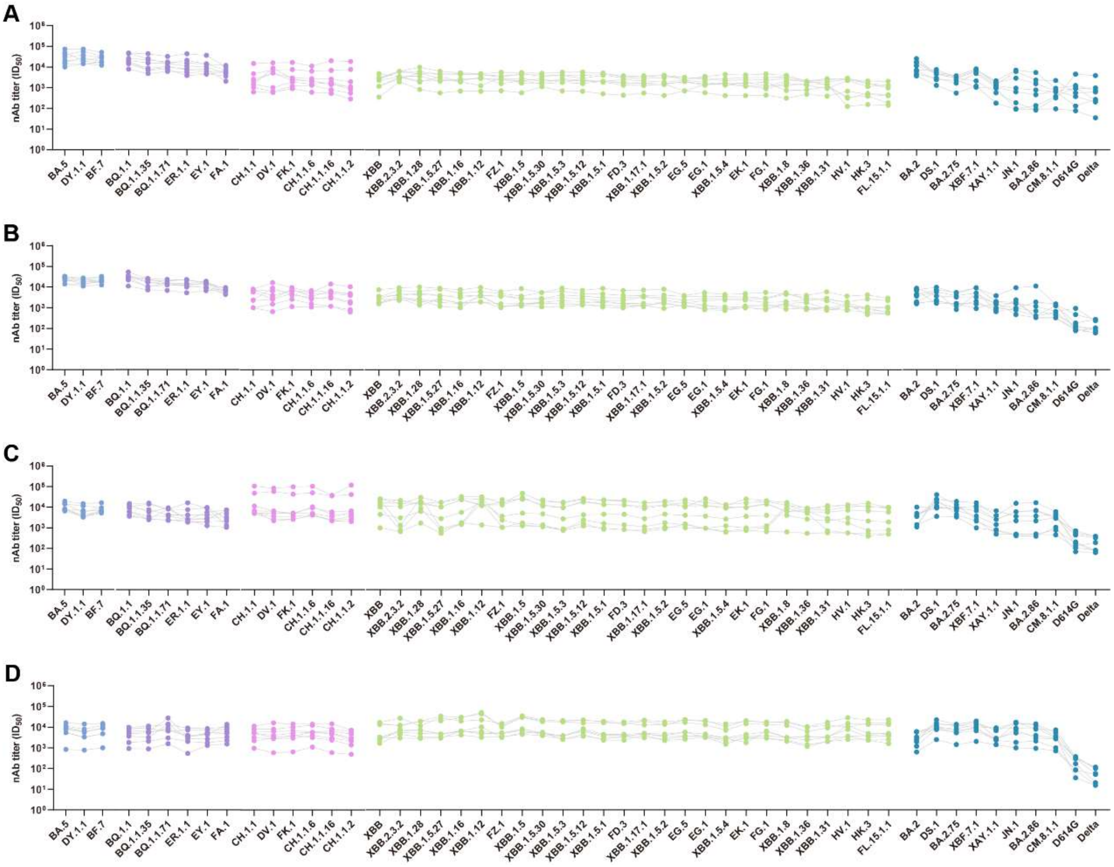

Figure S4 | Neutralizing antibody response profiles of immunized hamsters' sera, related to Figure 5.

Neutralization of 51 SARS-CoV-2 spike variants by hamsters' sera immunized with BA.5 (**A**, n=8), BQ.1.1 (**B**, n=8), CH.1.1 (**C**, n=8), and XBB.1.5 (**D**, n=8) antigens.

	NTD																														
	16	T19	R21	L24	P25	P26	A27	S50	Q52	H69	V70	V83	V127	G142	Y144	H146	K147	W152	E156	F157	R158	E180	Q183	I210	N211	L212	V213	L216	H245	A246	G252
D614G																															
Delta		R												D					G	del	del										
BA.2		I		del	del	del	S							D													G				
BA.2.75		I		del	del	del	S							D			E	R		L				V			G				
BA.2.86	ins MPLF	I	T	del	del	del	S	L		del	del		F	D	del					S	G				del	I	G	F	N	D	
JN.1	ins MPLF	I	T	del	del	del	S	L		del	del		F	D	del					S	G				del	I	G	F	N	D	
CH.1.1		I		del	del	del	S							D			E	R		L				V			G				
BA.5		I		del	del	del	S			del	del			D													G				
BQ.1.1		I		del	del	del	S			del	del			D													G				
FA.1		I		del	del	del	S			del	del			D	del												G				
XBB		I		del	del	del	S					A		D	del	Q							E				E				
XBB.1.5		I		del	del	del	S					A		D	del	Q							E				E				V
EG.5		I		del	del	del	S					A		D	del	Q							E				E				V
XBB.1.16		I		del	del	del	S					A		D	del	Q						V	E				E				V
HV.1		I		del	del	del	S		H			A		D	del	Q				L			E				E				V
HK.3		I		del	del	del	S		H			A		D	del	Q							E				E				V
FL.15.1.1		I		del	del	del	S					A		D	del	Q							E				E				V

	NTD	RBD																																
	G257	I332	G339	R346	K356	L368	S371	S373	S375	T376	R403	D405	R408	K417	N440	K444	V445	G446	N450	L452	L455	F456	N460	A475	S477	T478	N481	V483	E484	F486	F490	Q493	Q498	N501	Y505
D614G																																		
Delta																				R						K									
BA.2			D				F	P	F	A		N	S	N	K										N	K			A			R	R	Y	H
BA.2.75	S		S				F	P	F	A		N	S	N	K			S					K		N	K			A				R	Y	H
BA.2.86		V	H		T		F	P	F	A	K	N	S	N	K		H	S	D	W			K		N	K	K	del	K	P			R	Y	H
JN.1		V	H		T		F	P	F	A	K	N	S	N	K		H	S	D	W	S		K		N	K	K	del	K	P			R	Y	H
CH.1.1	S		H	T			F	P	F	A		N	S	N	K	T		S		R			K		N	K			A	S			R	Y	H
BA.5			D				F	P	F	A		N	S	N	K					R					N	K			A	V			R	Y	H
BQ.1.1			D	T			F	P	F	A		N	S	N	K	T				R			K		N	K			A	V			R	Y	H
FA.1			D	T			F	P	F	A		N	S	N	K	T				R			K		N	R			A	V			R	Y	H
XBB			H	T		I	F	P	F	A		N	S	N	K		P	S					K		N	K			A	S	S		R	Y	H
XBB.1.5			H	T		I	F	P	F	A		N	S	N	K		P	S					K		N	K			A	P	S		R	Y	H
EG.5			H	T		I	F	P	F	A		N	S	N	K		P	S				L	K		N	K			A	P	S		R	Y	H
XBB.1.16			H	T		I	F	P	F	A		N	S	N	K		P	S					K		N	R			A	P	S		R	Y	H
HV.1			H	T		I	F	P	F	A		N	S	N	K		P	S		R		L	K		N	K			A	P	S		R	Y	H
HK.3			H	T		I	F	P	F	A		N	S	N	K		P	S			F	L	K		N	K			A	P	S		R	Y	H
FL.15.1.1			H	T		I	F	P	F	A		N	S	N	K		P	S			F	L	K	V	N	K			A	P	S		R	Y	H

Figure S5 | Key amino acid mutations in NTD and RBD in highlighted variants, related to Figure 5.

NTD, N-terminal domain. RBD, receptor binding domain.

Supplementary Tables

Table S1 | Properties of 20 fluorescence proteins involved in this study, related to Figure 1A.

Fluorescence proteins	Color group	Ex-Max (λ)	Em-Max (λ)	Fluorescence channels		Extinction coefficient (M^{-1} cm^{-1})	Quantum yield (%)	Oligomerization	Brightness	pKa	Molecular weight (kDa)
				Ex (λ)	Em (λ)						
mTagBFP2	Blue	399	454	390-420	430-500	50,600	64.0	Monomer	32.38	2.7	26.7
mTurquoise2	Cyan	434	474	435-460	470-515	30,000	93.0	Monomer	27.90	3.1	26.9
mTFP1	Cyan	462	492	435-460	470-515	64,000	85.0	Monomer	54.40	4.3	26.9
mGreenLantern	Green	503	514	460-490	515-580	101,800	72.0	Monomer	73.30	5.6	26.8
StayGold	Green	496	515	460-490	515-580	159,000	93.0	Dimer	147.87	4.0	24.6
mNeonGreen	Green/Yellow	506	517	460-490	515-580	116,000	80.0	Monomer	92.80	5.7	26.6
mAmetrine	Long Stokes Shift	406	526	390-420	500-550	45,000	58.0	Monomer	26.10	6.0	26.8
LSSmOrange	Long Stokes Shift	437	572	435-460	570-620	52,000	45.0	Monomer	23.40	5.7	26.7
CyOFP1	Long Stokes Shift	497	589	490-515	570-620	40,000	76.0	Monomer	30.40	5.5	26.4
mCRISPRed	Long Stokes Shift	460	592	435-460	570-620	28,500	46.0	Monomer	13.11	2.1	26.6
mRuby3	Red	558	592	530-560	570-620	128,000	45.0	Monomer	57.60	4.8	26.6
mScarlet-I	Red	569	593	530-560	570-620	104,000	54.0	Monomer	56.16	5.4	26.4
LSSmScarlet	Long Stokes Shift	470	598	460-490	570-620	30,200	42.0	Monomer	12.68	5.8	26.3
mBeRFP	Long Stokes Shift	446	611	435-460	570-620	65,000	27.0	Monomer	17.55	5.6	26.4
hmKeima8.5	Long Stokes Shift	438	612	435-460	570-620	32,000	34.0	Monomer	10.88	5.3	25.1
emiRFP670	Near-infrared	642	670	615-645	655-760	87,400	14.0	Monomer	12.24	4.5	34.2
iRFP670	Near-infrared	643	670	615-645	655-760	114,000	11.0	Dimer	12.54	4.0	34.5
miRFP670nano	Near-infrared	645	670	615-645	655-760	95,000	10.8	Monomer	10.26	3.7	17.1
miRFP713	Near-infrared	690	713	650-675	685-760	99,000	7.0	Monomer	6.93	3.5	34.6
emiRFP703	Near-infrared	674	703	650-675	685-760	90,900	8.6	Monomer	7.82	4.5	34.2

Ex, excitation wavelength. Em, emission wavelength. Data were obtained from FPbase (https://www.fpbase.org).

Table S2 | Human sera used in this study, related to the right panel of Figure 4A.

ID	Gender	Age	SARS-CoV-2 vaccination status	With past SARS-CoV-2 infection
S001	male	41	3-dose of IV	no
S002	male	26	2-dose of IV	no
S003	female	36	3-dose of IV	yes
S004	female	42	3-dose of IV	yes
S005	female	46	3-dose of IV	yes
S006	female	42	3-dose of IV	yes
S007	female	30	3-dose of IV	yes
S008	male	46	3-dose of IV	yes
S009	female	54	3-dose of IV	yes
S010	female	37	3-dose of IV	yes
S011	female	27	3-dose of IV	yes
S012	female	32	3-dose of IV	yes
S013	female	39	3-dose of IV	yes
S014	female	30	3-dose of IV	yes
S015	male	45	3-dose of IV	yes
S016	female	41	3-dose of IV	yes
S017	female	38	3-dose of IV	yes

IV, inactivated vaccine.

Table S4 | Detailed neutralizing antibody response profiles of immunized hamsters' sera, related to Figure 5B.

	BA.5			BQ.1.1			CH.1.1			XBB.1.5		
	GMT	rNT	P value	GMT	rNT	*P* value	GMT	rNT	*P* value	GMT	rNT	*P* value
**BA.5**	26164	-	-	22948	0.863	>0.9999	10277	0.933	>0.9999	6492	0.601	>0.9999
**DY.1.1**	27885	1.066	>0.9999	17580	0.661	>0.9999	5630	0.511	>0.9999	4580	0.424	>0.9999
**BF.7**	22960	0.878	>0.9999	21502	0.809	>0.9999	7764	0.705	>0.9999	6461	0.599	>0.9999
**BQ.1.1**	19913	0.761	>0.9999	26583	-	-	7325	0.665	>0.9999	3869	0.358	0.8470
**BQ.1.1.35**	12273	0.469	>0.9999	14617	0.550	>0.9999	5176	0.470	>0.9999	3841	0.356	0.8866
**BQ.1.1.71**	11827	0.452	>0.9999	13601	0.512	>0.9999	4872	0.442	>0.9999	6690	0.620	>0.9999
**ER.1.1**	10298	0.394	>0.9999	12286	0.462	>0.9999	3641	0.331	>0.9999	2852	0.264	0.1044
**EY.1**	9115	0.348	>0.9999	11998	0.451	>0.9999	3643	0.331	>0.9999	3384	0.314	0.2241
**FA.1**	5376	0.205	>0.9999	6005	0.226	>0.9999	2959	0.269	>0.9999	4485	0.415	>0.9999
**CH.1.1**	2112	0.081	0.0038	3170	0.119	0.0181	11011	-	-	4022	0.373	>0.9999
**DV.1.1**	3056	0.117	0.1751	3454	0.130	0.0841	7762	0.705	>0.9999	3744	0.347	>0.9999
**FK.1**	2771	0.106	0.1079	4412	0.166	0.8957	7321	0.665	>0.9999	4263	0.395	>0.9999
**CH.1.1.6**	2281	0.087	0.0095	3024	0.114	0.0337	10142	0.921	>0.9999	4978	0.461	>0.9999
**CH.1.1.16**	2172	0.083	0.0070	3609	0.136	0.0789	5666	0.515	>0.9999	3975	0.368	>0.9999
**CH.1.1.2**	1541	0.059	<0.0001	2420	0.091	0.0008	7477	0.679	>0.9999	2541	0.235	0.0385
**XBB**	1705	0.065	0.0003	2562	0.096	0.0373	9481	0.861	>0.9999	4719	0.437	0.8091
**XBB.2.3.2**	3596	0.137	>0.9999	3851	0.145	>0.9999	3938	0.358	>0.9999	7381	0.684	>0.9999
**XBB.1.28**	3184	0.122	>0.9999	3277	0.123	0.9411	10601	0.963	>0.9999	7113	0.659	>0.9999
**XBB.1.5.27**	2583	0.099	0.4104	3162	0.119	0.7324	3418	0.310	>0.9999	7653	0.709	>0.9999
**XBB.1.16**	2436	0.093	0.3886	2451	0.092	0.0385	7036	0.639	>0.9999	9606	0.890	>0.9999
**XBB.1.12**	2423	0.093	0.2088	3603	0.136	>0.9999	12797	1.162	>0.9999	10377	0.961	>0.9999

	BA.5			BQ.1.1			CH.1.1			XBB.1.5		
	**GMT**	**rNT**	**P value**	**GMT**	**rNT**	***P* value**	**GMT**	**rNT**	***P* value**	**GMT**	**rNT**	***P* value**
**FZ.1**	2339	0.089	0.1969	2246	0.084	0.0082	5063	0.460	>0.9999	5905	0.547	>0.9999
**XBB.1.5**	2335	0.089	0.1969	2216	0.083	0.0038	7230	0.657	>0.9999	10795	-	-
**XBB.1.5.30**	2307	0.088	0.0572	2297	0.086	0.0102	4831	0.439	>0.9999	7793	0.722	>0.9999
**XBB.1.5.3**	2292	0.088	0.1220	2726	0.103	0.1857	3446	0.313	>0.9999	5906	0.547	>0.9999
**XBB.1.5.12**	2178	0.083	0.0536	2465	0.093	0.0455	6302	0.572	>0.9999	8473	0.785	>0.9999
**XBB.1.5.1**	2155	0.082	0.0412	2361	0.089	0.0223	4826	0.438	>0.9999	5868	0.544	>0.9999
**FD.3**	1804	0.069	0.0020	2067	0.078	0.001	4477	0.407	>0.9999	5739	0.532	>0.9999
**XBB.1.17.1**	1768	0.068	0.0014	2254	0.085	0.0065	3534	0.321	>0.9999	6808	0.631	>0.9999
**XBB.1.5.2**	1728	0.066	0.0008	2295	0.086	0.0157	4689	0.426	>0.9999	5410	0.501	>0.9999
**EG.5**	1705	0.065	0.0038	1818	0.068	<0.0001	4655	0.423	>0.9999	7042	0.652	>0.9999
**EG.1**	1626	0.062	0.0002	1780	0.067	<0.0001	4250	0.386	>0.9999	6247	0.579	>0.9999
**XBB.1.5.4**	1588	0.061	0.0002	1571	0.059	<0.0001	3224	0.293	>0.9999	3889	0.360	0.1235
**EK.1**	1564	0.060	<0.0001	1763	0.066	<0.0001	3913	0.355	>0.9999	5564	0.515	>0.9999
**FG.1**	1484	0.057	0.0001	1689	0.064	<0.0001	4417	0.401	>0.9999	6493	0.602	>0.9999
**XBB.1.8**	1480	0.057	0.0002	2119	0.080	0.0011	5739	0.521	>0.9999	4935	0.457	0.7726
**XBB.1.36**	1312	0.050	<0.0001	1679	0.063	<0.0001	4166	0.378	>0.9999	3451	0.320	0.0586
**XBB.1.31**	1266	0.048	<0.0001	1551	0.058	<0.0001	3043	0.276	>0.9999	4498	0.417	0.4353
**HV.1**	842	0.032	0.0283	1349	0.051	0.3114	2717	0.290	>0.9999	7330	0.679	>0.9999
**HK.3**	707	0.027	0.0250	1076	0.040	>0.9999	12714	0.238	>0.9999	5683	0.526	>0.9999
**FL.15.1.1**	571	0.022	0.0220	921	0.035	0.0076	7530	0.218	>0.9999	5653	0.524	>0.9999
**BA.2**	8812	0.337	>0.9999	3510	0.132	0.3481	4352	0.247	>0.9999	2500	0.232	0.0170
**DS.1**	3523	0.135	>0.9999	3789	0.143	<0.0001	1595	1.155	0.0315	8945	0.829	>0.9999
**BA.2.75**	2151	0.082	0.0385	2221	0.084	<0.0001	1922	0.684	0.2483	6428	0.595	>0.9999
**XBF.7.1**	3462	0.132	>0.9999	3274	0.123	<0.0001	214	0.395	0.0017	8570	0.794	>0.9999

	BA.5			BQ.1.1			CH.1.1			XBB.1.5		
	**GMT**	**rNT**	**P value**	**GMT**	**rNT**	***P* value**	**GMT**	**rNT**	***P* value**	**GMT**	**rNT**	***P* value**
**XAY.1.1.1**	899	0.034	<0.0001	1353	0.051	<0.0001	144	0.145	0.0011	3435	0.318	0.0699
**JN.1**	730	0.028	0.0014	1385	0.052	<0.0001	1997	0.181	0.3222	5978	0.554	>0.9999
**BA.2.86**	512	0.020	0.0051	821	0.031	<0.0001	2072	0.188	0.5292	4593	0.425	>0.9999
**CM.8.1.1**	518	0.020	<0.0001	659	0.025	<0.0001	1922	0.175	0.2483	2493	0.231	0.0026
**D614G**	544	0.021	<0.0001	165	0.006	<0.0001	214	0.019	0.0017	158	0.015	<0.0001
**Delta**	391	0.015	<0.0001	119	0.004	<0.0001	144	0.013	0.0011	43	0.004	<0.0001

GMT, geometric mean titer. rNT, mean relative nAb titer. P-values were calculated using two-tailed Wilcoxon signed-rank tests of paired samples.
